# Expanding anti-CD38 immunotherapy for lymphoid malignancies

**DOI:** 10.1186/s13046-022-02421-2

**Published:** 2022-06-28

**Authors:** Xu Wang, Xinfang Yu, Wei Li, Praveen Neeli, Ming Liu, Ling Li, Mingzhi Zhang, Xiaosheng Fang, Ken H. Young, Yong Li

**Affiliations:** 1 Department of Medicine, Baylor College of Medicine, Houston, TX USA; 2 National Clinical Research Center for Respiratory Disease, State Key Laboratory of Respiratory Disease, Guangzhou Institute of Respiratory Health, the First Affiliated Hospital of Guangzhou Medical University, Guangzhou, 510120 China; 3 Department of Oncology, Lymphoma Diagnosis and Treatment Center of Henan Province, the First Affiliated Hospital of Zhengzhou University, Zhengzhou, China; 4 Department of Pathology, Division of Hematopathology, Duke University Medical Center, Durham, NC USA; 5 Department of Hematology, Shandong Provincial Hospital, Shandong First Medical University, Jinan, Shandong China

**Keywords:** Multiple myeloma, Non-Hodgkin lymphoma, CD38, CAR T, Daratumumab, All-trans retinoic acid

## Abstract

**Background:**

Lymphoid neoplasms, including multiple myeloma (MM), non-Hodgkin lymphoma (NHL), and NK/T cell neoplasms, are a major cause of blood cancer morbidity and mortality. CD38 (cyclic ADP ribose hydrolase) is a transmembrane glycoprotein expressed on the surface of plasma cells and MM cells. The high expression of CD38 across MM and other lymphoid malignancies and its restricted expression in normal tissues make CD38 an attractive target for immunotherapy. CD38-targeting antibodies, like daratumumab, have been approved for the treatment of MM and tested against lymphoma and leukemia in multiple clinical trials.

**Methods:**

We generated chimeric antigen receptor (CAR) T cells targeting CD38 and tested its cytotoxicity against multiple CD38^high^ and CD38^low^ lymphoid cancer cells. We evaluated the synergistic effects of all-trans retinoic acid (ATRA) and CAR T cells or daratumumab against cancer cells and xenograft tumors.

**Results:**

CD38-CAR T cells dramatically inhibited the growth of CD38^high^ MM, mantle cell lymphoma (MCL), Waldenstrom’s macroglobulinemia (WM), T-cell acute lymphoblastic leukemia (T-ALL), and NK/T-cell lymphoma (NKTCL) in vitro and in mouse xenografts. ATRA elevated CD38 expression in multiple CD38^low^ cancer cells and enhanced the anti-tumor activity of daratumumab and CD38-CAR T cells in xenograft tumors.

**Conclusions:**

These findings may expand anti-CD38 immunotherapy to a broad spectrum of lymphoid malignancies and call for the incorporation of ATRA into daratumumab or other anti-CD38 immunological agents for cancer therapy.

**Supplementary Information:**

The online version contains supplementary material available at 10.1186/s13046-022-02421-2.

## Background

Lymphoid neoplasms, including multiple myeloma (MM), Hodgkin lymphoma, non-Hodgkin lymphoma (NHL), and NK/T cell neoplasms, remain one of the most significant health problems accounting for over 35,000 deaths in the United States in 2020 [1]. Although substantial progress for lymphoid malignancies treatment with conventional approaches (chemotherapy, surgery, radiation therapy, and targeted therapy) has been advanced during the past few decades, the majority of patients will suffer from disease relapse and die within the first decade after diagnosis [2, 3]. Until recently, the immunotherapy that utilizes and strengthens the power of patients' own immune systems to fight cancer has emerged as one of the most promising approaches to cancer treatment.

CD38, also known as cyclic ADP ribose hydrolase, is a cell surface glycoprotein of the ribosyl cyclase family that regulates cell migration, receptor-mediated adhesion, extracellular metabolites, and intracellular Ca^2+^ signaling transduction pathway [4, 5]. Generally, CD38 is expressed at high levels on MM cells and at relatively low levels on normal lymphoid and myeloid cells and some non-hematopoietic tissue [4, 6–8]. Importantly, the pluripotent hematopoietic stem cells that are crucial for long-term marrow recovery do not express CD38 [9], making CD38 an attractive therapeutic target. Daratumumab is a monoclonal antibody targeting CD38 in the treatment of MM that was approved by the FDA in 2015. Researchers are actively expanding the use of daratumumab for other CD38^+^ blood cancers, including acute lymphoblastic leukemia (ALL), natural killer/T-cell lymphoma (NKTCL), and acute myeloid leukemia [6]. A Phase 2 study of daratumumab in relapsed/refractory (R/R) NHL did not achieve satisfactory results [10].

A rapidly emerging immunotherapy approach utilizes gene transfer technologies to stably express chimeric antigen receptors (CARs) against a surface antigen on tumor cells. The CAR consists of a single-chain fragment variant (scFv) that acts as an extracellular antigen recognition domain derived from a specific antibody, a transmembrane domain anchored to the T cells, and T cell activation domains [11]. CAR T cells against CD19 antigen and B-cell maturation antigen (BCMA) have been approved to treat refractory diffuse large B-cell lymphoma (DLBCL), ALL, and MM [12–15]. In a preclinical study, CAR T cells against CD38 exhibit anti-tumor activities for MM cells and xenografts [16, 17].

In this project, we evaluated the feasibility of targeting CD38 in lymphoid malignancies. We first demonstrated that CD38-CAR T cells have robust anti-tumor function against CD38^high^ lymphoid cancer cells. We then identified that all-trans retinoic acid (ATRA) upregulates CD38 expression in CD38^low^ cancer cells and displays synergistic activity with CD38-CAR T cells and daratumumab. This work lends support to the use of ATRA and anti-CD38 immunotherapies against lymphoid cancers.

## Material and methods

### Generation of CD38-CAR T cells

Peripheral blood mononuclear cells (PBMCs) obtained from de-identified healthy donors at Gulf Coast Regional Blood Center (Houston, TX) were isolated from leukapheresis products by Ficoll-Paque (Cytiva, Marlborough, MA) gradient centrifugation. T cells were then negatively selected by the EasySep™ Human T Cell Isolation Kit (STEMCELL Technologies, Vancouver, Canada). These cells were activated by anti-CD3/CD28-coated beads (Life Technologies, Carlsbad, CA) at a cell-to-bead ratio of 1:3 with 200 U/ml of IL-2 (PeproTech, London, UK) in CTS™ OpTmizer™ T Cell Expansion SFM (Life Technologies). After 24–48 h of activation, cells were transduced by the addition of high-titer lentiviral particles expressing CD38-CAR, CD19-CAR, or green fluorescent protein (GFP). Transduced T cells were maintained at a concentration of 0.7 × 10^6^ cells/ml for 7–10 days by cell enumeration every 2–3 days. Finally, the T cells were induced to proliferate using a previously described rapid expansion protocol (REP) [18].

### Cell lines and reagents

All cell lines used in this study were listed in Supplemental Table 1. The MM cell lines (MM.1S, OPM-1, NCI-H929, RPMI-8226, KMS-12, and ANBL-6), Jurkat (T-ALL), and K562 (CML, the only myeloid cancer cell line) were purchased from the American Type Culture Collection (Manassas, VA). The Waldenstrom macroglobulinemia (WM) cell line RPCI-WM1 was obtained previously [19]. NKTCL cell lines KHYG-1, HANK-1, and SNK-6 were gifts from Dr. Javeed Iqbal, College of Medicine, University of Nebraska Medical Center. NKTCL cell lines YT and NK-YS were gifts from Dr. John Chan, Department of Pathology, City of Hope National Medical Center. The NKTCL, MCL, and MM cell lines NK-92, JeKo-1, Granta-519, Mino, SP-53, and MM.1R were gifts from Dr. Qing Yi, Center for Translational Research in Hematological Malignancies, Houston Methodist Hospital. The DLBCL cell line WSU (WSU-DLCL) and WILL-2 were gifts from Dr. Ken H. Young, Department of Pathology, Duke University Medical Center. MOLT-4 was obtained from Molecular and Cellular Biology Tissue Culture Core Laboratory, Baylor College of Medicine. Cells were cultured in their respective media with 10% FBS, 100 U/mL penicillin, and 100 mg/mL streptomycin. NK-92 cell lines were cultured with α-MEM with 0.2 mM inositol, 0.1 mM 2-mercaptoethanol, 0.02 mM folic acid, and 100 U/ml recombinant IL-2. KHYG-1, HANK-1, NK-YS, and SNK-6 were cultured with 20 U/ml, 10 U/ml, 100 U/ml, and 300 U/ml recombinant IL-2, respectively. All cell lines were cultured at 37˚C with 5% CO_2_ under humidified conditions. Chemical reagents dimethyl sulfoxide and retinoic acid (cat. # R2625) were purchased from Sigma-Aldrich (St. Louis, MO). U73122 (cat. # S8011) and selumetinib (cat. # S1008) were obtained from Selleck Chemicals (Houston, TX). Ipatasertib (cat. #A3006), PF-431396 (cat. #A8692), and saracatinib (cat. #A2133) were obtained from ApexBio (Houston, TX). Proteome Profiler Human Phospho-Kinase Array Kit (cat. #ARY003C) was obtained from R & D Systems (Minneapolis, MN). Antibodies against β-actin (cat. #A5316) were obtained from Sigma-Aldrich. Anti-CD38 (cat. # ab108403) antibody was obtained from Abcam (Cambridge, UK). Anti-PYK2 (cat. #3292S) and anti-p-PYK2 (cat. #3291S) were obtained from Cell Signaling Technology (Danvers, MA).

### Xenograft models

Six to eight-week-old NOD.Cg-Prkdcscid Il2^rgtm1Wjl/SzJ^ (NSG) mice were purchased from Jackson Laboratory (Bar Harbor, ME) and maintained at Baylor College of Medicine Animal Facility. All procedures were carried out with IACUC approval at Baylor College of Medicine. For the MM model, 1 × 10^6^ firefly luciferase (fLuc)-expressing OPM-1 was intravenously injected into NSG mice. One week later, a single injection of 5 × 10^6^ of CD38-CAR T cells or non-transduced T cells were injected into NSG mice through the tail vein. For the MCL and WM model, 0.8 × 10^6^ fLuc-JeKo-1 or fLuc-RPCI-WM1 was injected into NSG mice, which were then treated with 5 × 10^6^ of CD38-CAR T cells after 1 week. For the T-ALL model, 5 × 10^6^ fLuc-MOLT-4 was intravenously injected into NSG mice, which were treated with a single injection of 8 × 10^6^ of CD38-CAR T cells. For the therapeutic NKTCL model, 5 × 10^6^ YT was subcutaneously injected into NSG mice, which were treated with two injections of 8 × 10^6^ of CD38-CAR T cells at day 18 and 20. For the prophylactic NKTL model, 5 × 10^6^ YT was subcutaneously injected into NSG mice, which were treated with a single injection of 5 × 10^6^ of CD38-CAR T cells on day 10. For the SP-53-CAR T model, 3 × 10^6^ SP-53 was subcutaneously injected into NSG mice, which were treated with two doses of 8 × 10^6^ of CD38-CAR T cells after 5 days (with or without intraperitoneal ATRA treatment). For the SP-53-daratumumab model, 3 × 10^6^ SP-53 was subcutaneously injected into NSG mice, which were treated with two doses of 1 × 10^7^ of PBMCs (NK-cell-enriched) after 8 days (with ATRA and/or daratumumab) [20]. Tumor burden was monitored either with a vernier caliper every 2–3 days or using an IVIS Imaging System (Caliper Life Sciences, Waltham, MA) that recorded bioluminescence from mice injected intraperitoneally with 150 mg/kg of D-luciferin (Gold Technology, St. Louis, MO) at indicated time points. Tumor volume was calculated according to the formula: tumor volume (mm^3^) = (length × width × width/2), where length is the longest diameter and width is the shortest. Living Image software (PerkinElmer, Waltham, MA) was used to visualize and calculate total luminescence.

### Western blot analysis

Cells were lysed by 1 × RIPA lysis buffer (Thermo Fisher Scientific, Waltham, MA) with 1% SDS and protease and phosphatase inhibitors (Thermo Fisher Scientific) and then collected and centrifuged at 12,000 rpm for 15 min in 4˚C. The supernatant was measured with the BCA protein assay reagent (Thermo Fisher Scientific). Lysates were denatured in Laemmli sample buffer (Bio-Rad, Hercules, CA) and resolved by Tris–glycine SDS-PAGE (4–20% polyacrylamide, Mini-PROTEAN Precast Gels, Bio-Rad). After transferring to the polyvinyl difluoride membrane, the membrane was blocked with 5% non-fat dry milk in 0.1% TBS-Tween-20 for 2 h and incubated with the primary antibody at 4˚C overnight. HRP-conjugated anti-rabbit or anti-mouse IgG (Cell Signaling Technology) was used as the secondary antibody. Immunoreactive protein was visualized with the enhanced chemiluminescent (ECL) western blotting substrate (Thermo Fisher Scientific).

### RNA preparation and qRT-PCR

Total RNA was extracted using Trizol (Thermo Fisher Scientific) and reverse-transcribed into cDNA with the iScript™ cDNA synthesis kit according to the manufacturer's instructions (Bio-Rad). Gene expression levels were quantified by qRT-PCR performed on a QuantStudio 7 Pro qRT-PCR system. The qRT-PCR analysis was performed using primers for each gene, and the results were normalized to Beta-2-microglobulin (β2M) transcript levels. The difference in fold expression was calculated using the ΔΔCT method. Primers used against each gene were validated for specificity using BLAST and melting curve analysis.

### Luciferase-based cytotoxicity assay in vitro

To measure cytotoxicity, 2–5 × 10^4^ cells (MM.1S, OPM-1, HANK-1, MOLT-4, RPCI-WM1, JeKo-1, Granta-519, K562) expressing fLuc were co-cultured with T cells in a round-bottom 96-well plate (Corning, Corning, NY) at an indicated effector: target (E:T) ratio for 4 or 20 h at 37 °C. D-Luciferin (Thermo Fisher Scientific) was then added, and luminescence was measured with a Synergy HTX Multi-Mode Reader (BioTek Instruments, Winooski, VT). The lysis of tumor cells was calculated as [1-(target cells with CD38-CAR T cells/target cells with control T cells)] × 100%. Assays were performed in triplicate.

### Flow cytometry

Anti-human CD3 (cat. #300308), CD28 (cat. #302907), CD38 (cat. #303526), CD95 (cat. #305607), PD-1 (cat. #329937), PD-L1 (cat. #329708), CTLA-4 (cat. #349906), CCR7 (cat. #353210), and CD45RA (cat. #304154) from BioLegend, San Diego, CA, and TIM-3 (cat. #17–3109-42) and CD107a (cat. #A15729) from Thermo Fisher Scientific were used to stain cells. Biotin-Protein L (cat. # M00097, GenScript, Piscataway, NJ) was used for CAR detection [21]. CellTrace™ Far Red Cell Proliferation Kit (Thermo Fisher Scientific) was used to monitor T cell proliferation. All flow cytometry data were obtained in BD Accuri™ C6 Plus, BD LSRFortessa (BD Biosciences, San Jose, CA), or CyTek™ NL-3000 (Cytek Biosciences, Fremont, CA) and analyzed with FlowJo software (FlowJo).

### Cytokine release in vitro

T cells and tumor cells were incubated at a 2:1 ratio for 20 h. Supernatants were harvested and subjected to ELISA for cytokine production according to the manufacturer’s instructions (R & D System).

### Histology immunohistochemistry analyses (IHC)

Tissue samples were subjected to hematoxylin and eosin (H&E) staining or IHC staining by Human Tissue Acquisition and Pathology (HTAP) Core Lab in the Baylor College of Medicine following the manufacturer’s protocol, as described previously [22].

### Statistics

All statistical analyses were performed using the Prism v7.0 program (GraphPad Software, San Diego, CA). Data are presented as means ± standard deviation (SD). Comparisons between two groups were analyzed using the Student’s t-test, whereas that for three or more groups were tested using the two-way repeated-measures analysis of variance. Kaplan–Meier analysis and the log-rank test (Mantel-Cox) were used for survival analysis. A probability value of *p* ≤ 0.05 was considered statistically significant.

## Results

### Generation of CD38-CAR T cells using lentiviral gene transfer

**Fig. 1:**
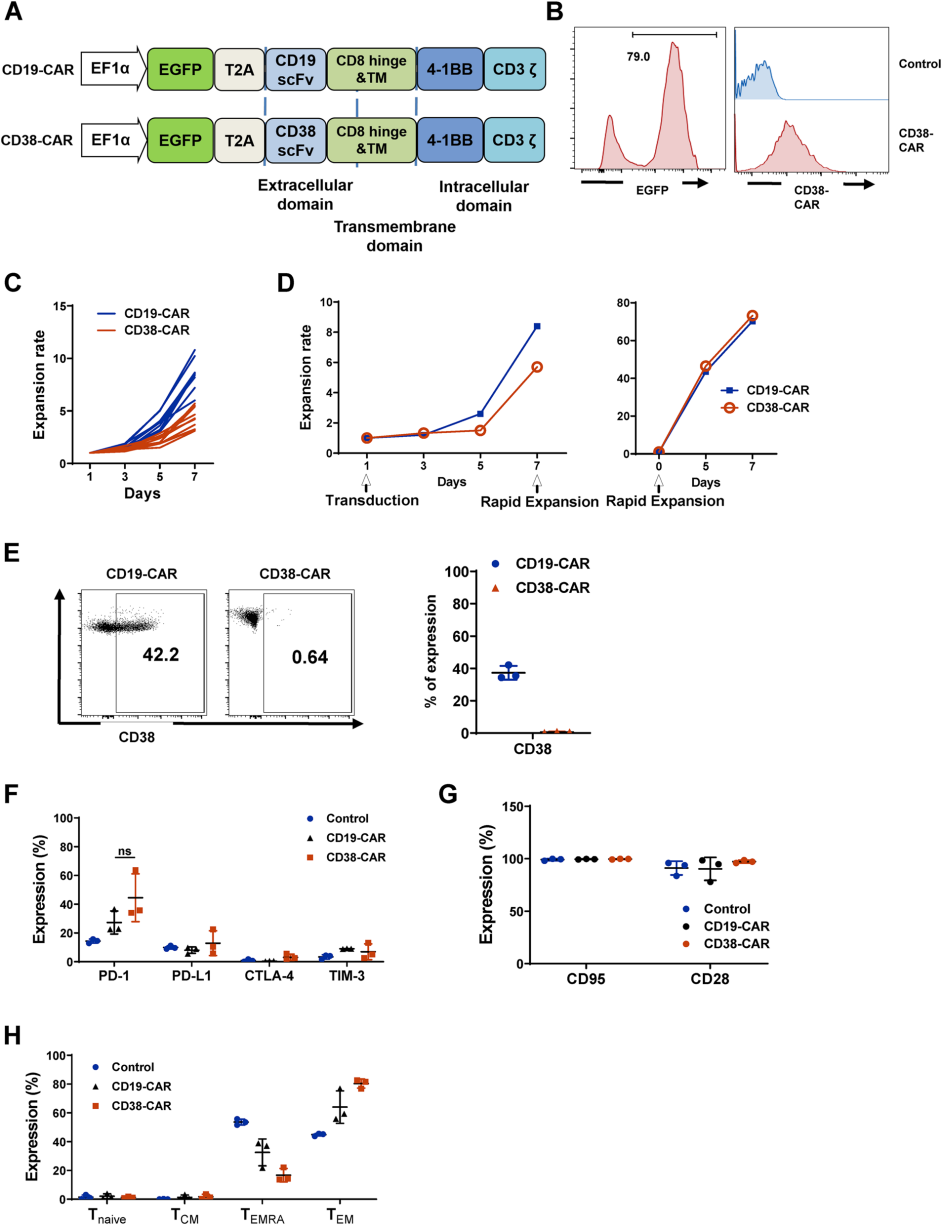
CD38-CAR T cells generation and characterization. **A** Schematic of the CAR constructs. **B** The expression levels of EGFP and CD38-CAR on transduced T cells as determined. Vector without CAR insertion was used as controls. **C** Expansion of T cells transduced with CD19-CAR and CD38-CAR in vitro for 7 days (*n* = 10, healthy donors). (D) Representative graph of REP applied on the expansion of T cells transduced with CD19-CAR and CD38-CAR in vitro for 7 days. **E** CD38 expression level in CD38-CAR T cells and CD19-CAR T cells (control) (*n* = 3). **F–H** Expression of checkpoint molecules (PD-1, PD-L1, CTLA-4, and TIM-3); Expression of T cell activation markers (CD95 and CD28; *n* = 3); The percentage of T cells that were positive for memory cell markers (T_naive_: CD45RA^+^CCR7^+^; T_CM_: CD45RA^−^CCR7^+^; T_EMRA_: CD45RA^+^CCR7^−^; T_EM_: CD45RA^−^CCR7^−^; *n* = 3)

We constructed the CD38-CAR by utilizing anti-CD38 scFv linked to 4-1BB and CD3ζ chain stimulation signaling domains in a third-generation self-inactivating lentiviral vector with EF1α promoter [17]. The scFv is based on a published monoclonal antibody with affinity similar to daratumumab [17]. To evaluate the transduction efficiency, we incorporated enhanced green fluorescent protein (EGFP) fluorescent reporter gene ahead of P2A self-cleaving peptides at the N-terminal of the CAR sequence (Fig. 1A). As a control, CD19-CAR was generated based on the same backbone as CD38-CAR [22]. The high transduction efficiency in human primary T cells was measured by flow cytometry through the detection of both EGFP and anti-CD38 scFv (Fig. 1B). CD38-CAR T cells quickly expanded after initial activation by anti-CD3/CD28 beads during the first 7 days (*n* = 10) (Fig. 1C). Due to CD38 expression on normal T cells [16, 17], the expansion of CD38-CAR T cells was slower than that of CD19-CAR T cells using our T cell culturing system (Fig. 1C). Therefore, a REP that utilized irradiated feeder cells was applied to expand CAR-T cells after 7 days of transduction to achieve 80–100 fold proliferation for primary CD3^+^ T cells within 7–10 days (Fig. 1D). After 10 days of culture of transduced T cells, a loss of CD38 expression in T cells was observed (Fig. 1E). To determine whether the loss of CD38 affected T cell function, we characterized T cell activation markers and checkpoint molecules after 10 days of priming activation. Empty vector-transduced T cells and CD19-CAR T cells were used as controls. CD38-CAR T cells displayed a similar level of activation markers and checkpoint molecules, indicating that they were fully functional (Fig. 1F–H). These data are in agreement with a previous report [17, 18] and imply that CD38-CAR T cells acquire a CD38^low^ phenotype and rapidly expand 80–100-fold in cell numbers that can be used in the clinic.

### CD38-CAR T cells exhibit anti-MM function in vitro* and *in vivo

**Fig. 2:**
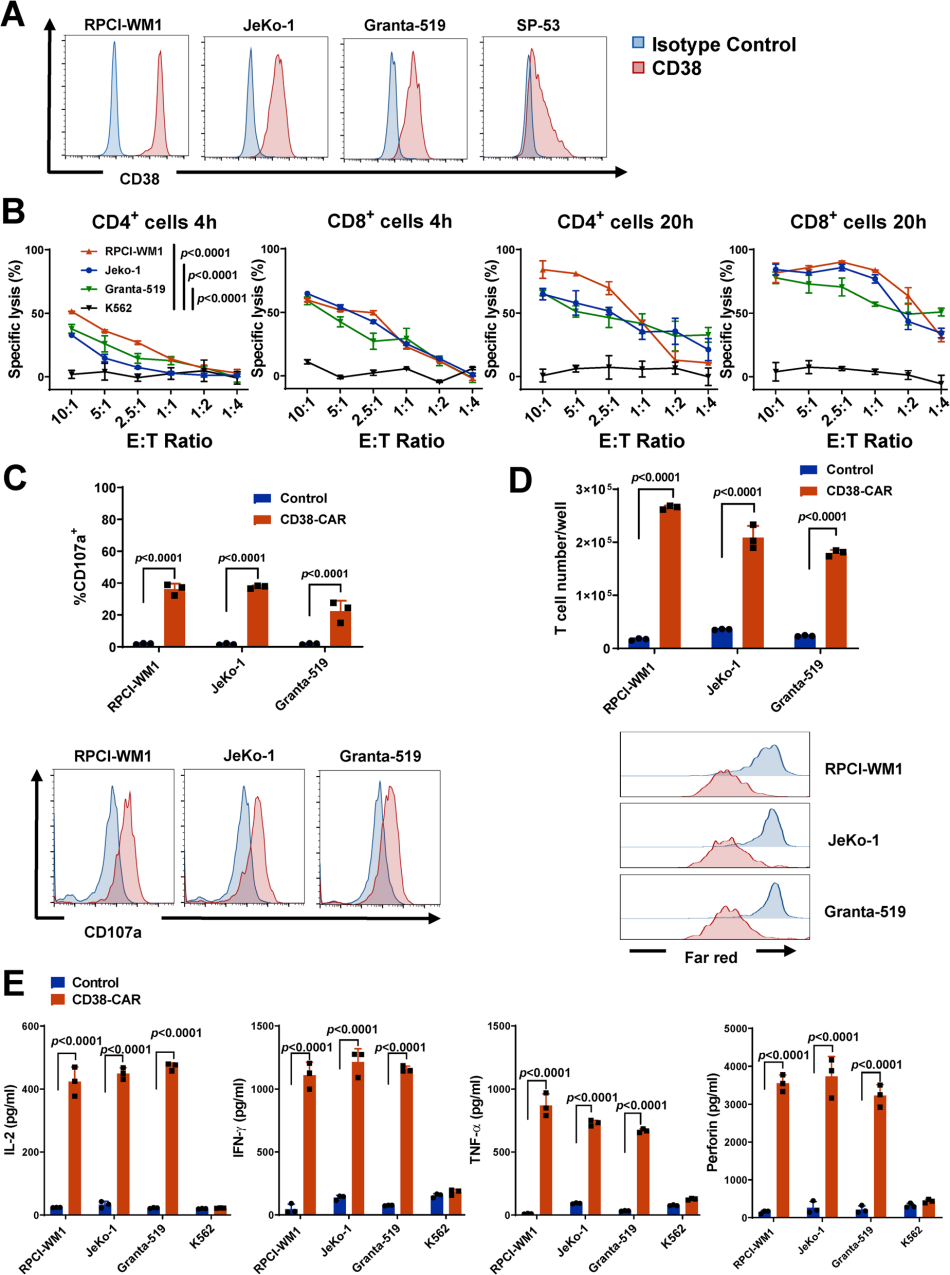
Potent effector function of CD38-CAR T cells against WM and MCL in vitro. **A** CD38 expression in WM (RPCI-WM1) and MCL (JeKo-1, Granta-519, and SP-53) cell lines by flow cytometry. **B** The cytotoxic activity of CD4^+^ and CD8^+^ CD38-CAR T cells. T cells were co-cultured with WM and MCL cells and K562 (negative control) with stably expressed fLuc for 4 and 20 h, respectively, at various effector (E):target (T) ratios. **C** Evaluation of CD107a expression by flow cytometry. Effector cells were co-cultured with target cells for 6 h at 2:1 E:T ratio. **D** Proliferation assessed by absolute cell number and CellTrace™ far-red proliferation dilution after 5 days of co-culture of effector and target cells. Assays were performed with effector cells and irradiated target cells at 1:2 E:T ratio without the addition of exogenous cytokines. **E** Secretion of IL-2, IFN- γ, TNF-α, and perforin from effector cells by ELISA. Assays were performed using supernatants obtained after a 20-h co-culture of effector and target cells at a 2:1 E:T ratio

The surface expression level of CD38 in MM cell lines MM.1S, NCI-H929, OPM-1, RPMI-8226, KMS-12, and ANBL-6 was detected using flow cytometry. Other than KMS-12, all lines expressed a relatively high level of CD38 (Fig. S1A). We made recombinant cells lines (MM.1S, OPM-1, and K562) expressing an exogenous fLuc. K562 is a chronic myelogenous leukemia (CML) cell line expressing no CD38 as a negative control. We co-cultured CD4^+^ and CD8^+^ CD38-CAR T cells with fLuc-MM.1S, fLuc-OPM-1, and fLuc-K562 at various E:T ratios for 4 h or 20 h, respectively. Both CD4^+^ and CD8^+^ CAR T cells exerted specific and high-efficient cytolysis activities against MM.1S and OPM-1, achieving as high as 80% specific lysis by CD8^+^ CAR T cells after 20 h (Fig. S1B). No specific lysis was found for K562 cells (Fig. S1B) and the K562 dataset was used throughout the study (Fig. 2; Fig. 4). The expression of CD107a, a T cell granulation marker, was significantly elevated in CD38-CAR T cells, but not in control T cells co-cultured with 3 MM lines (MM.1S, OPM-1, NCI-H929; Fig. S1C). In addition, CD38-CAR T cells underwent multiple divisions within 5 days when co-cultured with each MM line (Fig. S1D). A significant amount of IL-2, IFN-γ, TNF-α, and perforin was released by CD38-CAR T cells co-cultured with MM cells (Fig. S1E). By contrast, cytokine and perforin production was quite limited in control T cells (Fig. S1E). Lastly, we injected 1 × 10^6^ fLuc-OPM-1 cells intravenously into the NSG mice. Seven days after tumor inoculation, mice were injected intravenously with a single dose of 5 × 10^6^ CD38-CAR T cells or non-transduced T cells (Fig. S2A). As shown in Figs. S2B and S2C, CD38-CAR T cells mediated significant regression of MM xenografts. This regression led to a significant survival advantage compared with that of mice with no treatment or with non-transduced T cells (Fig. S2D).

### CD38-CAR T cells eradicate B-cell lymphoid cancer cells in vitro and in vivo

**Fig. 3:**
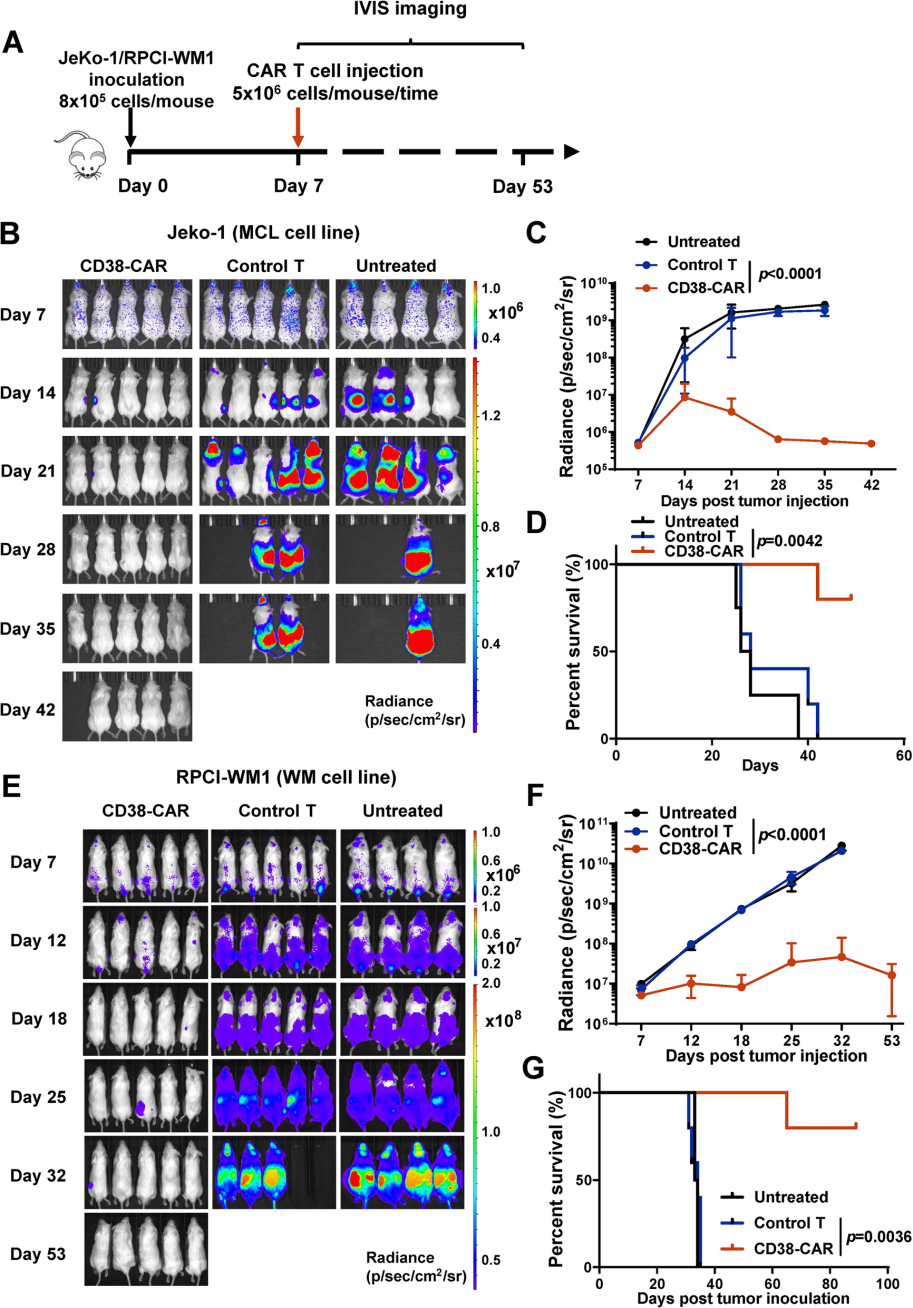
Potent effector function of CD38-CAR T cells against WM and MCL in vivo. **A** Schematic of CAR T-cell treatment protocol. NSG mice were intravenously injected with 8 × 10^5^ fLuc-transduced MCL cells (JeKo-1). After 7 days, the mice were treated with 5 × 10^6^ CD38-CAR T cells or non-transduced T cells (control T cells), or remained untreated (*n* = 5 or 4). **B** A serial of bioluminescent imaging results showing lymphoma progression/regression in mice. **C** Kinetics of lymphoma progression, measured by bioluminescent imaging, in mice treated with CD38-CAR T cells, control T cells, or untreated. **D** Survival curve for each experimental group. **E** A serial of representative bioluminescent results showing WM tumor progression/regression in mice. **F** Kinetics of tumor progression, measured by bioluminescent imaging, in mice treated with CD38-CAR T cells, control T cells, or remained untreated. **G** Survival curve for each experimental group

We analyzed CD38 expression levels on MCL and WM cell lines by flow cytometry. Three MCL cell lines (JeKo-1, Granta-519, and SP-53) and one WM cell line (RPCI-WM1) expressed various levels of CD38 (Fig. 2A). To determine the cytotoxicity of CD38-CAR T cells towards MCL and WM cells, we co-cultured CD4^+^ or CD8^+^ CD38-CAR T cells with CD38^high^ JeKo-1, Granta-519, and RPCI-WM1 cells for 4 h or 20 h. At a 10:1 E:T ratio, both CD4^+^ and CD8^+^ CD38-CAR T cells lysed MCL or WM cells, achieving ~ 80% specific lysis by CD8^+^ CAR T cells after 20 h (Fig. 2B). To further characterize the cytolytic activity of CD38-CAR T cells, we assessed CD107a expression. High CD107a expression was only observed on CD38-CAR T cells, but not on control T cells, when they were co-cultured with CD38^high^ lymphoma cells (Fig. 2C). When CD38^high^ MCL and WM cells were co-cultured, CD38-CAR T cells were more proliferative than control T cells (Fig. 2D). In addition, significant amounts of TNF-α, IFN-γ, IL-2, and perforin were secreted by CD38-CAR T cells (Fig. 2E). Only minimal amounts of cytokines were produced by CD38-CAR T cells co-cultured with the CD38-negative K562 cells or by control T cells co-cultured with the CD38^high^ lymphoma cells (Fig. 2E). To test the efficacy of CD38-CAR T cells against MCL or WM in vivo, we used immunodeficient NGS xenograft models. We injected 8 × 10^5^ fLuc-JeKo-1 or fLuc-RPCI-WM1 cells intravenously into the NSG mice. Seven days after tumor inoculation, mice were injected intravenously with a single dose of 5 × 10^6^ CD38-CAR T cells or non-transduced T cells as the control (Fig. 3A). Tumor burden was monitored by in vivo bioluminescence imaging beginning on day 7. As shown in Fig. 3, CD38-CAR T cells mediated significant regression of xenografts of either MCL or WM cells. This regression led to a significant survival advantage for CD38-CAR T-cell-treated mice compared with the control group (Fig. 3D and G).

### Targeting CD38 on non-B cell neoplasms in vitro and in vivo

**Fig. 4:**
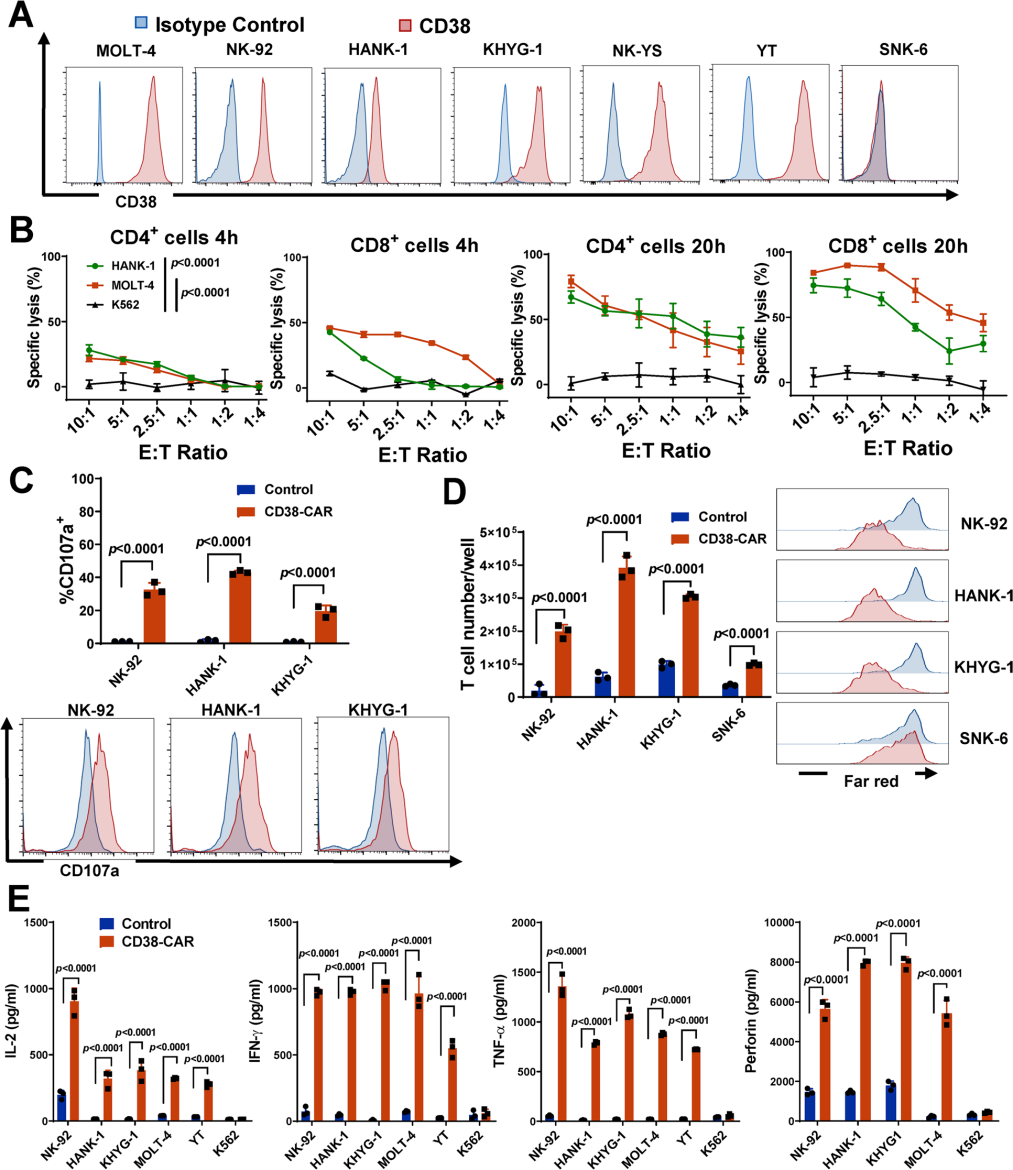
Potent effector function of CD38-CAR T cells against T-ALL and NKTCL in vitro. **A** CD38 expression in T-ALL (MOLT-4) and NKTCL (NK-92, HANK-1, KHYG-1, NK-YS, YT, SNK-6) cell lines by flow cytometry. **B** The cytotoxic activity of CD4^+^ and CD8^+^ CD38-CAR T cells. T cells were co-cultured with T-ALL and NKTCL cells and K562 (negative control) with stably expressed fLuc for 4 and 20 h, respectively, at various E:T ratios. **C** Evaluation of CD107a expression by flow cytometry. Effector cells were co-cultured with target cells for 6 h at a 2:1 E:T ratio. **D** Proliferation assessed by absolute cell number and CellTrace™ far-red proliferation dilution after 5 days of co-culture of effector and target cells. Assays were performed with effector cells and irradiated target cells at a 1:2 E:T ratio without the addition of exogenous cytokines. **E** Secretion of IL-2, IFN- γ, TNF- α, and perforin from effector cells by ELISA. Assays were performed using supernatants obtained after a 20 h co-culture of effector and target cells at a 2:1 E:T ratio

CD38 is broadly expressed in non-B cell neoplasms, so next, we investigated the anti-tumor effects of CD38-CAR-T cells in T-ALL and NKTCL models. CD38 was highly expressed on NKTCL cell lines (NK-92, HANK-1, KHYG-1, NK-YS, and YT) and the T-ALL cell line (MOLT-4). We performed the fLuc-based cytotoxicity assay against both T-ALL and NKTCL cell lines (MOLT-4 and HANK-1). After 4 or 20 h of co-culture at different E:T ratios, both CD4^+^ and CD8^+^ CD38-CAR T cells showed robust cytotoxicity against MOLT-4 or HANK-1 (Fig. 4B). Furthermore, the cytolytic activity correlated with elevated CD107a expression (Fig. 4C), increased T-cell proliferation (Fig. 4D), and augmented cytokine production (Fig. 4E).

**Fig. 5:**
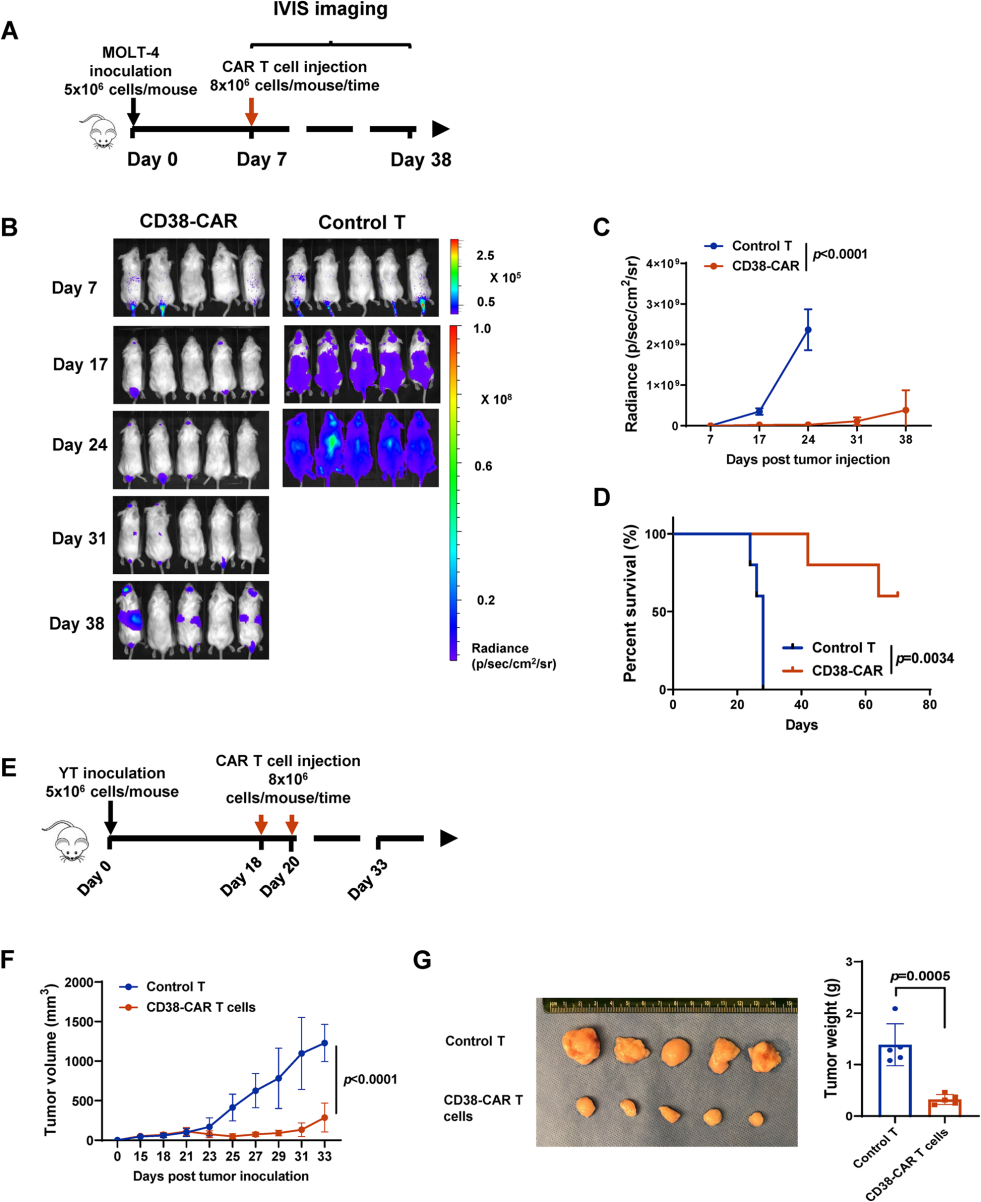
Potent effector function of CD38-CAR T cells against T-ALL and NKTCL in vivo. **A** Schematic of CAR T cells treatment protocol. NSG mice were intravenously injected with 5 × 10^6^ fLuc-transduced T-ALL cells (MOLT-4). After 7 days, the mice were treated with 8 × 10^6^ CD38-CAR T cells or control T cells (*n* = 5). **B** A serial of representative bioluminescent imaging showing leukemia progression/regression in mice. **C** Kinetics of leukemia progression in mice treated with CD38-CAR T cells or control T cells measured by bioluminescent imaging. **D** Survival curve for each experimental group. **E** Schematic of CAR T cells treatment protocol. NSG mice were subcutaneously injected with 5 × 10^6^ NKTCL cells (YT). After 18 or 20 days, the mice were treated with 8 × 10^6^ CD38-CAR T cells or control T cells (*n* = 5). **F** Mean tumor growth kinetics with the treatment of CD38-CAR T cells or control T cells. **G** Images of CD38-CAR T cells or control T cells-treated tumor harvested on 33^rd^ day after implantation. Tumor weight **G** was measured per group (*n* = 5)

Next, the effects of CD38-CAR T cells on T-ALL and NKTCL in vivo were evaluated. NSG mice were inoculated intravenously with fLuc-MOLT-4 cells (Fig. 5). Seven days after tumor injection, mice received CD38-CAR-T cells, a single dose of 8 × 10^6^ CD38-CAR T cells, and non-transduced T cells (Fig. 5B). As shown in Fig. 4B-D, CD38-CAR-T cells significantly reduced tumor growth and improved animal survival. For NKTCL, YT tumor cells were implanted subcutaneously into NSG mice. When the tumor size approached ~ 100 mm^3^ (18–20 days), two doses of 8 × 10^6^ CD38-CAR T cells were delivered intravenously (Fig. 5E). As shown in Fig. 5F and G, the CD38-CAR T cells significantly reduced tumor burden and slowed disease progression (all mice had residual tumors at the end of the study). When treatments were administered before tumors were established (on day 10 post tumor cell inoculation), 4 of 5 mice were tumor-free upon CD38-CAR T-cell treatment (Figs. S3A and S3B).

### Evaluation of the role of ATRA on CD38 enhancement in CD38^low^ lymphoid cancer cells in vitro

**Fig. 6:**
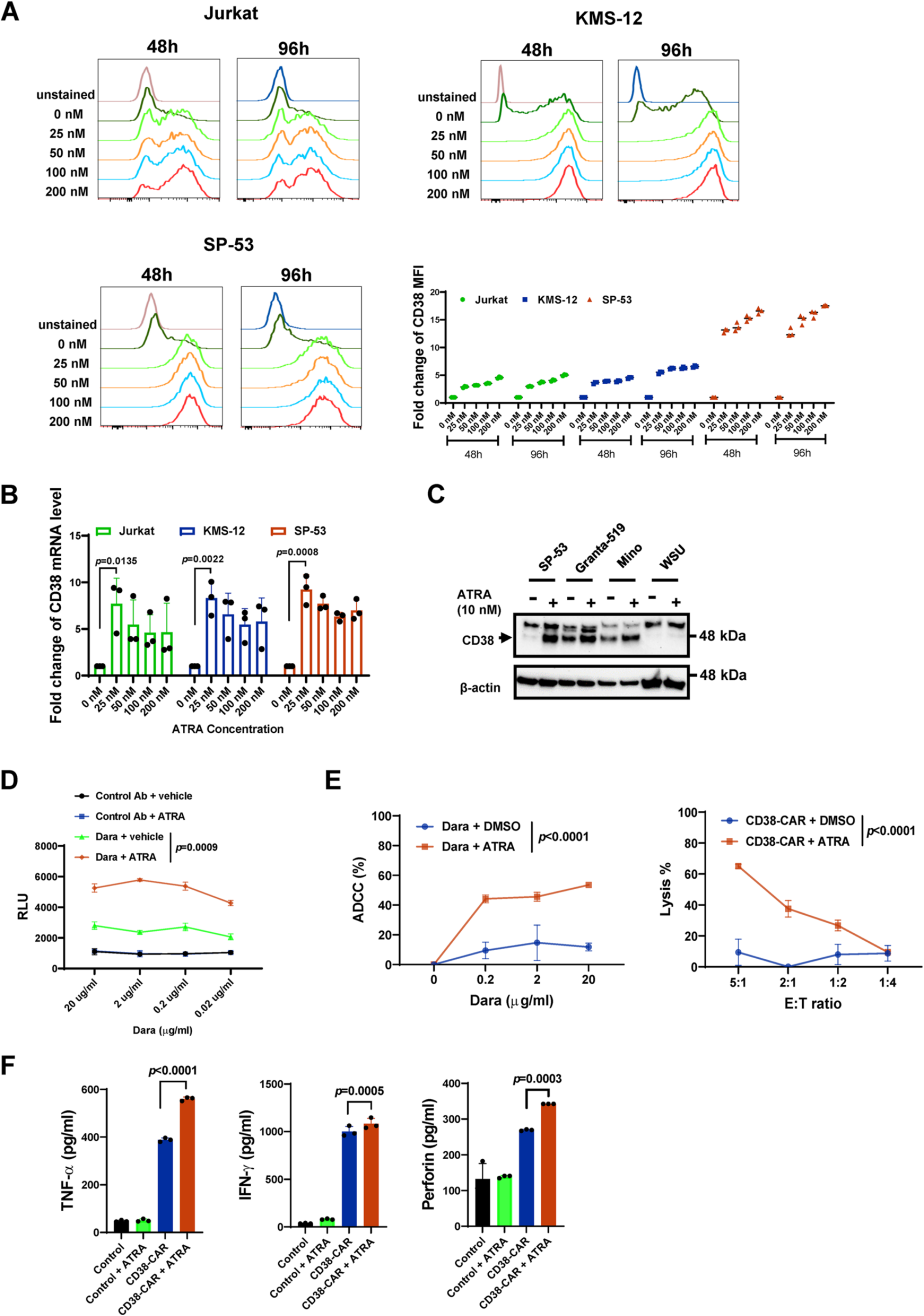
Upregulation of CD38 by ATRA in lymphoid cancer cells. **A** CD38 expression in the CD38^low^ cell lines with or without ATRA treatment by flow cytometry. Jurkat, SP-53, or KMS-12 cells were treated with vehicle or ATRA with indicated final concentration for 48 or 96 h; the fold change of median fluorescence intensity (MFI) of CD38 in each cell line was blotted. **B** mRNA levels of CD38 in Jurkat, SP-53, or KMS-12 with or without treatment of ATRA with indicated concentration for 24 h. **C** The CD38 expression levels were evaluated by immunoblotting. NHL cell lines (SP-53, Granta-519, Mino, or WSU) were treated with ATRA (10 nM) for 48 h. **D** ADCC reporter cells responses to daratumumab were dependent on CD38 levels. SP-53 cells were pre-treated with ATRA (10 nM) for 48 h, followed by the ADCC reporter assay using Jurkat/NFAT-Luc/FcγRIIIa effector cells. **E** ATRA enhanced CD38-based ADCC or CAR T cell cytotoxicity in vitro. In the left panel, SP-53 cells were pre-treated with ATRA (10 nM) for 48 h followed by the treatment with daratumumab and IgG1 isotype antibody with PBMCs for 6 h; in the right panel, the cytotoxic activity of CD38-CAR T cells was measured co-culturing with SP-53 cells with or without ATRA (10 nM) pre-treatment for 6 h. **F** Secretion of IFN- γ, TNF- α, and perforin from T cells by ELISA. Assays were performed uisng supernatants obtained after a 20-h co-culture of T cells (CD38-CAR T cells and control T cells) and SP-53 cells with or without ATRA pre-treatment at a 2:1 E:T ratio

Previously, ATRA was shown to enhance CD38 expression and improve the cytotoxic effects of daratumumab on MM cells [20]. We examined the effects of various doses of ATRA on CD38 expression in lymphoid cancer cells (Fig. S4A-C). ATRA drastically upregulated CD38 expression at protein and mRNA levels in CD38^low^ Jurkat (T-ALL) and SP-53 (MCL) cells (Fig. 6A and B). CD38 was also upregulated by ATRA in CD38^low^ KMS-12 (MM) cells [20] (Fig. 6A-B). The impact of ATRA on CD38 upregulation was not as remarkable for CD38^high^ MM and MCL cells or 2 DLBCL lines (Fig. 6C and Fig. S4A-C). To a lesser extent than ATRA, other retinoids also elevated the expression of CD38 in SP-53 MCL cells (Fig. S5). We profiled the expression of CD27, a memory B-cell marker, in either CD38^low^ (SP-53) or CD38^high^ (Mino, Granta-519, and JeKo-1) MCL cell lines upon ATRA treatment. CD27 expression levels were upregulated by ATRA in all cell lines, indicating that ATRA drives B-cell differentiation towards a memory phenotype (Fig. S4D). To further determine whether the level of cell surface CD38 increases after ATRA treatment in CD38^low^ cells, ATRA- or vehicle-treated target cells were tested following daratumumab treatment using antibody-dependent cellular cytotoxicity (ADCC) reporter assay. ADCC bioassay effector cells are engineered to express the FcγRIIIa receptor and an NFAT response element driving the expression of firefly luciferase (NFAT-RE). The more interactions between an antibody and FcγRIIIa receptor, the stronger the luminance signal that will be observed. SP-53 cells, an MCL cell line with the highest response to ATRA treatment, were used as target cells. A higher, dose-dependent reporter signal was produced when the Jurkat effector cells were co-incubated with ATRA-pretreated SP-53 and daratumumab than without ATRA-pretreated SP-53 and daratumumab (Fig. 6D).

We determined whether CD38 upregulation by ATRA increased the anti-CD38 immunotherapies in vitro. SP-53 cells pre-treated with ATRA for 48 h were incubated with PBMCs plus daratumumab or CD38-CAR T cells. We observed stronger cytotoxicity against ATRA-treated cells mediated by either daratumumab and CD38-CAR T cells than untreated SP-53 cells (Fig. 6E). The increase in CAR T-cell-mediated cytotoxicity was accompanied by elevated production of cytokines like TNF-α, IFN-γ, and perforin (Fig. 6F). Collectively, these data support the potential clinical utility of ATRA with daratumumab or CD38-CAR T cells against CD38^low^ lymphoid cancer cells, especially MCL cells.

### PYK2 activation is required for ATRA-induced CD38 upregulation

**Fig. 7:**
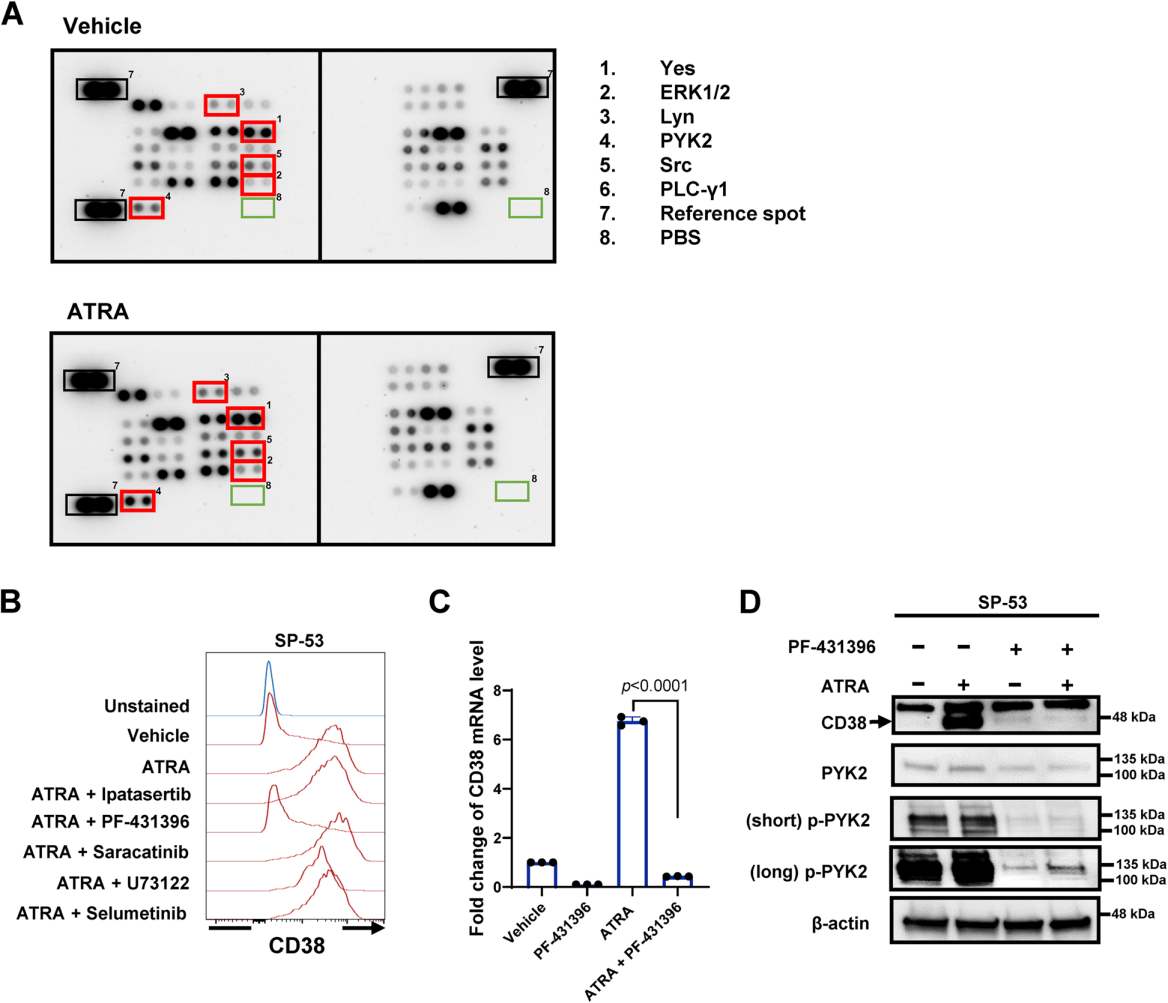
Activation of PYK2 is required for ATRA-mediated CD38 upregulation. **A** Kinase profiling of SP-53 cells treated with ATRA. SP-53 cells were treated with vehicle or ATRA (10 nM). 1–6, kinases; 7, reference spot; 8, negative control. The fold of phosphorylation upon ATRA over vehicle was shown on the right. **B** CD38 expression in SP-53 cells treated with ATRA or kinase inhibitors. SP-53 cells were treated with ATRA (10 nM) and/or kinase inhibitors for 48 h and subjected to flow cytometry with an anti-CD38 antibody. **C** mRNA levels of CD38 in SP-53 with or without treatment of ATRA (10 nM) or PF-431396. **D** SP-53 cells were treated with ATRA (10 nM) or PF-431396 for 48 h; CD38, PYK2, and p-PYK2 expression were evaluated by immunoblotting

We investigated the mechanism underlying ATRA-induced CD38 expression. Multiple kinases, such as protein kinase C (PKC) and phospholipase C gamma (PLC-γ), are reported to regulate CD38 expression [23, 24]. We performed a phospho-kinase array screen to detect phosphorylation of 43 human kinases in SP-53 cells treated with ATRA. Upon ATRA treatment, the phosphorylation levels of 6 kinases were elevated (Fig. 7A). Among them, Yes, ERK1/2, Lyn, and PYK2 are reported to be activated by ATRA in myeloid cells or myeloid neoplastic cells [25–27]. We then treated SP-53 cells with inhibitors to these kinases along with ATRA. PF-431396 targeting PYK2 significantly reversed ATRA-induced CD38 expression, whereas inhibitors to the others did not (Fig. 7B). Inhibition of PYK2 significantly decreased the CD38 mRNA levels that were induced by ATRA (Fig. 7C). We then performed western blot analyses of PYK2 phosphorylation at Y402 in response to ATRA and PYK2 inhibitor in SP-53 cells. ATRA induced high PYK2 Y402 phosphorylation and CD38 upregulation, which was inhibited by PF-431396 (Fig. 7D). As noted previously, ATRA upregulated CD38 moderately in CD38^high^ Mino and Granta-519 cells (Fig. 6C), yet PF-431396 reversed the upregulation only in Mino cells but not in Granta-519 cells (Fig. S4E). These results suggest that activation of PYK2 was required for ATRA-induced CD38 upregulation in some but not all MCL cells.

### ATRA improves the activity of CD38-CAR T cells and daratumumab in vivo

**Fig. 8:**
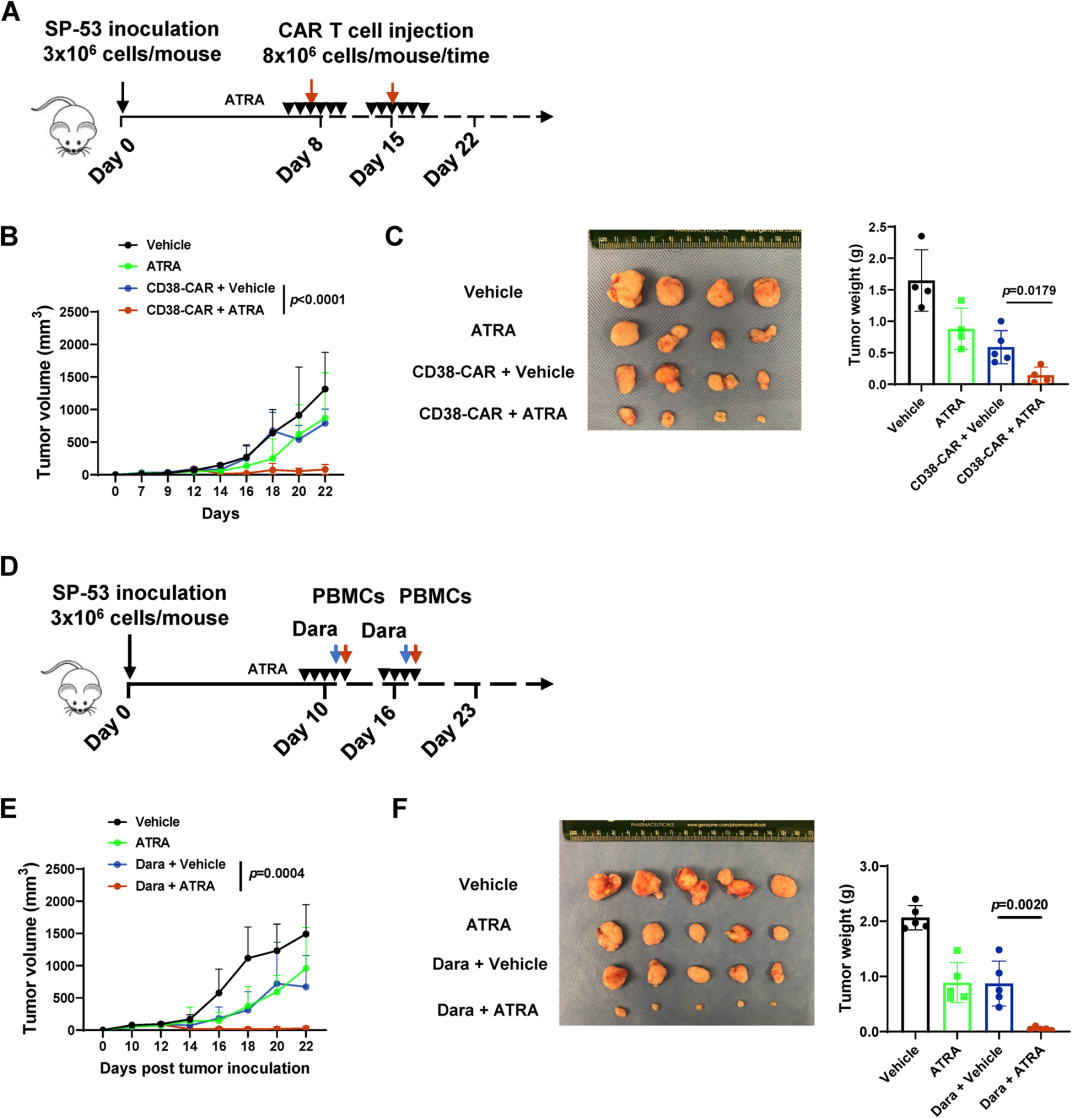
ATRA improves the efficacy of anti-CD38-based immunotherapy in a CD38^low^ MCL xenograft model. **A** Schematic of CAR T cells treatment protocol. NSG mice were subcutaneously injected with 3 × 10^6^ MCL cells (SP-53). At the indicated time points, the mice were treated with the vehicle, ATRA (10 mg/kg), CD38-CAR T cells, or CD38-CAR T cells plus ATRA (10 mg/kg; *n* = 4 or 5). **B** Mean tumor growth kinetics in different groups. **C** Images of tumors from indicated treatment groups were harvested on the 22^nd^ day after implantation. Tumor weight was measured (*n* = 4 or 5) per group. **D** Schematic of CAR T cells treatment protocol. NSG mice were subcutaneously injected with 3 × 10^6^ MCL cells (SP-53). At the indicated point, the mice were treated with the vehicle, ATRA (10 mg/kg), daratumumab with NK-enriched PBMCs, or daratumumab with NK-enriched PBMCs plus ATRA (10 mg/kg; *n* = 5). **E** Mean tumor growth kinetics in different groups. **F** Images of tumors from indicated treatment groups harvested on the 22^nd^ day after implantation. Tumor weight was measured (*n* = 5) per group

Finally, we investigated the potential anti-cancer benefit of ATRA in combination with CD38-CAR T cells or daratumumab. NSG mice that were treated with a series of doses of ATRA and 2 doses of CAR T cells were inoculated subcutaneously with SP-53 tumor cells (Fig. 8A). The combination significantly reduced tumor burden and disease progression, whereas the single agents only moderately did so (Fig. 8B and C). We then assessed the effects of ATRA and daratumumab in animals treated with multiple doses of ATRA and two doses of the antibody and NK cell-enriched PBMCs [20] (Fig. 8D). As shown in Fig. 8E and F, ATRA drastically improved the anti-tumor effect of daratumumab, whereas ATRA or daratumumab alone had only moderate efficacy. Notably, increased CD38 expression by ATRA was observed with immunohistochemistry staining (IHC) in SP-53 tumors (Figs. S6A and S6B). These data support the finding that ATRA improves the efficacy of anti-CD38-based immunotherapy against CD38^low^ lymphoid cancer cells by CD38 upregulation.

## Discussion

Unprecedented successes of CD19- and BCMA-specific CAR T cells in treating lymphoid malignancies have been seen in the last decade. CD38 is an attractive immunotherapy target for MM, as it is highly and ubiquitously expressed in MM cells and virtually absent in physiological organs [8]. Importantly, the pluripotent hematopoietic precursor cells that are crucial for a long-term marrow recovery do not express CD38 (CD34^+^CD38^−^) [9]. Daratumumab is a successful therapeutic monoclonal antibody targeting CD38 in the treatment of MM, which was approved by the FDA in 2015 [28]. In addition to MM, CD38 has also been found to express in some lymphoid cancers, but the expression is not uniform in WM [29], MCL [30], T-ALL [31, 32], and NKTCL [33–35]. A recent clinical trial tested the therapeutic efficacy of daratumumab against several NHLs, including DLBCL, follicular lymphoma (FL), and MCL [10]. Patients had an inadequate response to daratumumab, resulting in early termination of the trial [10]. The median percentage of CD38 expression for all three study cohorts (DLBCL, FL, MCL) was 70%, with the lowest value in the MCL cohort. It is notable that CD38 expression in relapsed NHL patients was lower than that in MM patients (> 90%) [36]. These data suggest that better approaches targeting CD38 against lymphoid cancers are needed.

We constructed CD38-CAR T cells and evaluated their therapeutic potential against lymphoid malignancies in the present study. CD38-CAR T cells displayed significant cytotoxic activity against cultured CD38^high^ MM, MCL, WM, T-ALL, and NKTCL cells, causing regression of established xenograft tumors from these cells and prolonged animal survival. However, these CAR T cells were less effective against CD38^low^ lymphoid cancer cells. ATRA is a compound used to treat acute promyelocytic leukemia [37]. As ATRA elevates CD38 expression and enhances the therapeutic benefit of daratumumab against MM cell lines, primary MM cells, and MM tumors in a humanized MM mouse model patients [20], we evaluated the potential of ATRA in CD38 upregulation in CD38^low^ lymphoid cancer cells. ATRA augmented the expression of CD38 in CD38^low^ T-ALL and MCL cell lines, most likely through the activation of the PYK2 kinase. Pharmacological inhibition of PYK2 completely repressed CD38 upregulation in MCL SP-53 cells induced by ATRA. We then examined the combination of ATRA with CD38-CAR T cells or daratumumab against the CD38^low^ SP-53 cells in mouse xenografts. ATRA induced CD38 expression within the xenograft tumors, and both combinations exhibited more robust anti-tumor activities than the monotherapies. These results imply that anti-CD38 immunotherapies, either CAR T cells or antibodies, are effective for cancers with high CD38 expression. The toxicity of ATRA alone [37] or in combination with arsenic trioxide [38] or anti-CD38 antibody [39] is acceptable. Thus, our data broaden the potential clinical use of anti-CD38 immunotherapies to treat CD38^high^ lymphoid cancer cells and support the combinations with ATRA to treat CD38^low^ cancers that are sensitive to ATRA.

A limitation of this study is the lack of demonstration of tumor cell killing by autologous CAR T cells with or without ATRA. DLBCL is the most common type of NHL with multiple cell surface markers (CD20, CD19, and CD22) for immunotherapeutic targeting. ATRA only moderately augments the expression of CD38 in WILL-2 and WSU DLBCL cells (Fig. S4C). WILL-2 was an isolated CD20^−^ cell clone from a CD20^+^ DLBCL patient treated with the anti-CD20 antibody rituximab [40]. Likely, anti-CD38 immunotherapies with ATRA may not be the next best option for rituximab-resistant DLBCL. A recent clinical trial tested the efficacy and safety of daratumumab combined with ATRA in R/R MM patients, yet the addition of ATRA and intensification of daratumumab had limited activity [39]. Thus, caution must be exercised when evaluating the clinical benefit of anti-CD38 and ATRA to patients with lymphoid neoplasms, particularly for R/R patients.

## Conclusion

This work is the first report concerning CD38-CAR T cells redirected to multiple lymphoid malignancies (MM, WM, MCL, T-ALL, and NKTCL) cells and/or xenograft tumors. Furthermore, the addition of ATRA sensitizes the response of CD38^low^ cancer cells to either CD38-CAR T cells or anti-CD38 antibodies. The potent cancer cell-eliminating ability of CD38-redirected CAR T cells and daratumumab armed with ATRA supports the conclusion of promising therapeutic options for lymphoid malignancies.

## Supplementary Information

**Additionalfile 1:** **Supplemental Figure S1.** Anti-myelomafunction of CD38-CAR T cells *in vitro. *(A) CD38 expression in MM cell lines (MM.1S, NCI-H929, OPM-1, RPMI-8226, KMS-12, and ANBL-6) measured by flow cytometry. (B) Thecytotoxic activity of CD4^+^ and CD8^+^ CD38-CAR T cells. T cells were co-cultured with MM cells and K562 (negative control) with stably expressed fLuc for 4 and 20 h, respectively, at various ratios of E:T. (C) Evaluation of CD107a expression by flow cytometry. Effector cells were co-cultured with target cells for 6 h at 2:1 E:T ratio. (D) Proliferation assessed by absolute cell number and CellTrace™ far-red proliferation dilution after a 5-day co-culture of effector and target cells. Assays were performed with effector cells and irradiated target cells at 1:2 E:T ratio without the addition of exogenous cytokines. (E)Secretion of IL-2, IFN-γ, TNF-α, and perforin from effector cells by ELISA. Assays were performed in supernatants obtained after a 20-h co-culture of effector and target cells at a 2:1 E:T ratio. **Supplemental Figure S2.** Anti-myeloma function of CD38-CAR T cells *invivo. *(A) Schematic of CAR T cells treatment protocol. NSG mice were intravenously injected with 1 × 10^6^ fLuc-transduced MM cells (OPM-1). After 7 days, the mice were treated with 5×10^6^ of CD38-CAR T cells, control T cells, or remained untreated (*n* = 5 or 4). (B) A serial of representative bioluminescent imaging showing myeloma progression/regression in mice. (C) Kinetics of myeloma progression in mice treated with CD38-CAR T cells, control T cells, or remained untreated measured by bioluminescent imaging. (D) Survival curve for each experimental group. **Supplemental Figure S3.** CD38-CAR T cells inhibit NKTCL growth in the prophylactic xenograftmodel. (A) Schematic of CAR T cells treatment protocol. NSG mice were subcutaneously injected with 5×10^6^ NKTCL cells (YT). After 10 days, the mice weretreated with 5×10^6^ of CD38-CAR T cells or control T cells (*n*= 5). (B) Mean tumor growth kinetics for each treatment group. **Supplemental FigureS4.** ATRA enhances the CD38 expression in MM, MCL, and DLBCL. (A–C) CD38 expression in MM, MCL, or DLBCL with or without ATRA (10 nM) treatment by flow cytometry. (D) CD27 expression in MCL cell lines with or without ATRA (10 nM) treatment (JeKo-1,Granta-519, Mino and SP-53) by flow cytometry. (E) Mino and Granta-519 were treated with ATRA (10 nM) or PF-431396 for 48 hours. CD38 expression were evaluated by immunoblotting. **Supplemental Figure S5.** Retinoids enhance the CD38 expression invariable degrees. (A) SP-53 cells were treated with adapalene, tazarotene,acitretin, isotretinoin, or the vehicle control ranging from 10 to 200 nM for 24 or 48 h. (B) The fold change of median fluorescence intensity (MFI) of CD38 in SP-53 cells after treatment of ATRA (10 nM) or four other retinoids for 24 or 48 h. **Supplemental Figure S6.** Hematoxylin and eosin (H&E) and immunohistochemistry (IHC) staining for tumors from mice with ATRA or anti-CD38-based immunotherapy. (A) Representative IHC images of indicated treatment SP-53 tumors for CD38. Scalebars, 100 μm; H&E staining of SP-53 tumors on day 22. Scale bars, 100 μm. (B) Representative IHC images of indicated treatment SP-53 tumors for CD38. Scale bars, 100 μm; H&E staining of SP-53 tumors on day 22. Scale bars, 100μm. **Supplemental Table 1.** Cell lines used in this study.

## Data Availability

All data and materials supporting the conclusion of this study have been included within the article and the supplemental data.

## Abbreviations

CD38: Cyclic ADP ribose hydrolase
MM: Multiple myeloma
CAR: Chimeric antigen receptor
NHL: Non-Hodgkin lymphoma
ATRA: All-trans retinoic acid
WM: Waldenstrom’s macroglobulinemia (WM)
T-ALL: T-cell acute lymphoblastic leukemia (T-ALL)
NKTCL: NK/T-cell lymphoma
MCL: Mantle cell lymphoma
DLBCL: Diffuse large B-cell lymphoma
FL: Follicular lymphoma
scFv: Single-chain fragment variant
BCMA: B-cell maturation antigen
PBMCs: Peripheral blood mononuclear Cells
PYK2: Protein-tyrosine kinase 2-β
NSG: NOD.Cg-Prkdcscid Il2^rgtm1Wjl/SzJ^
fLuc: Firefly luciferase
RIPA: Radioimmunoprecipitation assay
IHC: Immunohistochemistry
EGFP: Enhanced green fluorescent protein
REP: Rapid expansion protocol
ADCC: Antibody-dependent cellular cytotoxicity
NFAT: Nuclear factor of activated T cells
