# Supplementary material for: Expanding anti-CD38 immunotherapy for lymphoid malignancies
Source: J Exp Clin Cancer Res. 2022 Jun 28;41:210. doi: 10.1186/s13046-022-02421-2 (PMC9237984; doi:10.1186/s13046-022-02421-2)
Supplement: Supplementary file 1 — Additionalfile 1: Supplemental Figure S1. Anti-myelomafunction of CD38-CAR T cells in vitro. (A) CD38 expression in MM cell lines (MM.1S, NCI-H929, OPM-1, RPMI-8226, KMS-12, and ANBL-6) measured by flow cytometry. (B) Thecytotoxic activity of CD4+ and CD8+ CD38-CAR T cells. T cells were co-cultured with MM cells and K562 (negative control) with stably expressed fLuc for 4 and 20 h, respectively, at various ratios of E:T. (C) Evaluation of CD107a expression by flow cytometry. Effector cells were co-cultured with target cells for 6 h at 2:1 E:T ratio. (D) Proliferation assessed by absolute cell number and CellTrace™ far-red proliferation dilution after a 5-day co-culture of effector and target cells. Assays were performed with effector cells and irradiated target cells at 1:2 E:T ratio without the addition of exogenous cytokines. (E)Secretion of IL-2, IFN-γ, TNF-α, and perforin from effector cells by ELISA. Assays were performed in supernatants obtained after a 20-h co-culture of effector and target cells at a 2:1 E:T ratio. Supplemental Figure S2. Anti-myeloma function of CD38-CAR T cells invivo. (A) Schematic of CAR T cells treatment protocol. NSG mice were intravenously injected with 1 × 10^6^ fLuc-transduced MM cells (OPM-1). After 7 days, the mice were treated with 5×10^6^ of CD38-CAR T cells, control T cells, or remained untreated (n = 5 or 4). (B) A serial of representative bioluminescent imaging showing myeloma progression/regression in mice. (C) Kinetics of myeloma progression in mice treated with CD38-CAR T cells, control T cells, or remained untreated measured by bioluminescent imaging. (D) Survival curve for each experimental group. Supplemental Figure S3. CD38-CAR T cells inhibit NKTCL growth in the prophylactic xenograftmodel. (A) Schematic of CAR T cells treatment protocol. NSG mice were subcutaneously injected with 5×10^6^ NKTCL cells (YT). After 10 days, the mice weretreated with 5×10^6^ of CD38-CAR T cells or control T cells (n= 5). (B) Mean tumor growth kin [file 13046_2022_2421_MOESM1_ESM.pdf]

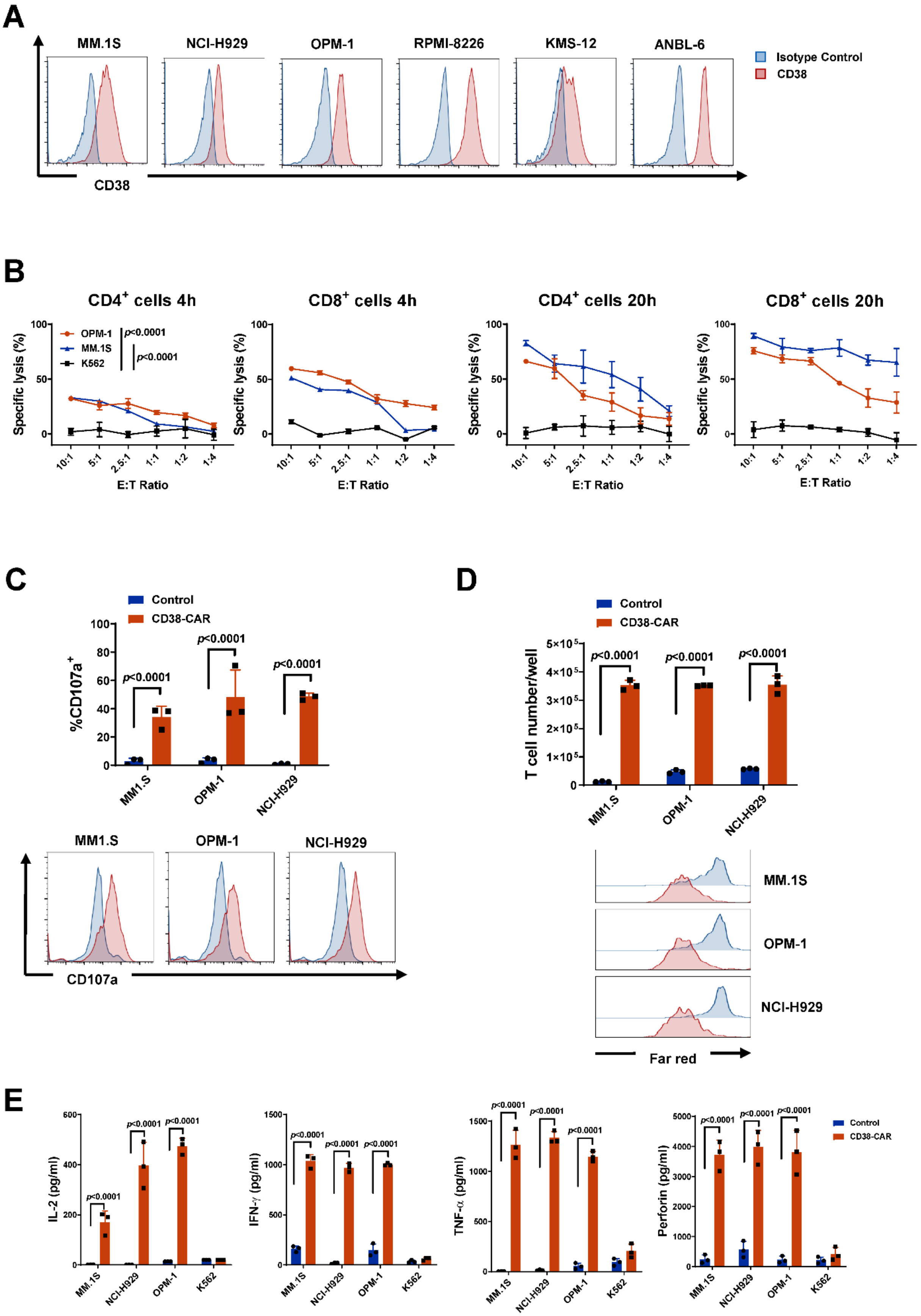
A
MM.1S
NCI-H929
OPM-1
RPMI-8226
KMS-12
ANBL-6
Isotype Control
CD38
CD38
B
CD4+ cells 4h
CD8+ cells 4h
CD4+ cells 20h
CD8+ cells 20h
OPM-1
MM.1S
K562
p<0.0001
p<0.0001
Specific lysis (%)
E:T Ratio
10:1
5:1
2.5:1
1:1
1:2
1:4
C
Control
CD38-CAR
%CD107a+
p<0.0001
MM1.S
OPM-1
NCI-H929
CD107a
D
Control
CD38-CAR
T cell number/well
p<0.0001
MM.1S
OPM-1
NCI-H929
Far red
E
IL-2 (pg/ml)
IFN-γ (pg/ml)
TNF-α (pg/ml)
Perforin (pg/ml)
p<0.0001
MM.1S
NCI-H929
OPM-1
K562
Control
CD38-CAR

Supplemental Figure S1. Anti-myeloma function of CD38-CAR T cells *in vitro.*

(A) CD38 expression in MM cell lines (MM.1S, NCI-H929, OPM-1, RPMI-8226, KMS-12, ANBL-6) measured by flow cytometry. (B) The cytotoxic activity of $CD4^+$ and $CD8^+$ CD38-CAR T cells. T cells were co-cultured with MM cells and K562 (negative control) with stably expressed fLuc for 4 and 20 h, respectively, at various ratios of E:T. (C) Evaluation of CD107a expression by flow cytometry. Effector cells were co-cultured with target cells for 6 h at 2:1 E:T ratio. (D) Proliferation assessed by absolute cell number and CellTrace™ far-red proliferation dilution after a 5-day co-culture of effector and target cells. Assays were performed with effector cells and irradiated target cells at 1:2 E:T ratio without the addition of exogenous cytokines. (E) Secretion of IL-2, IFN-γ, TNF-α, and perforin from effector cells by ELISA. Assays were performed in supernatants obtained after a 20-h co-culture of effector and target cells at a 2:1 E:T ratio.

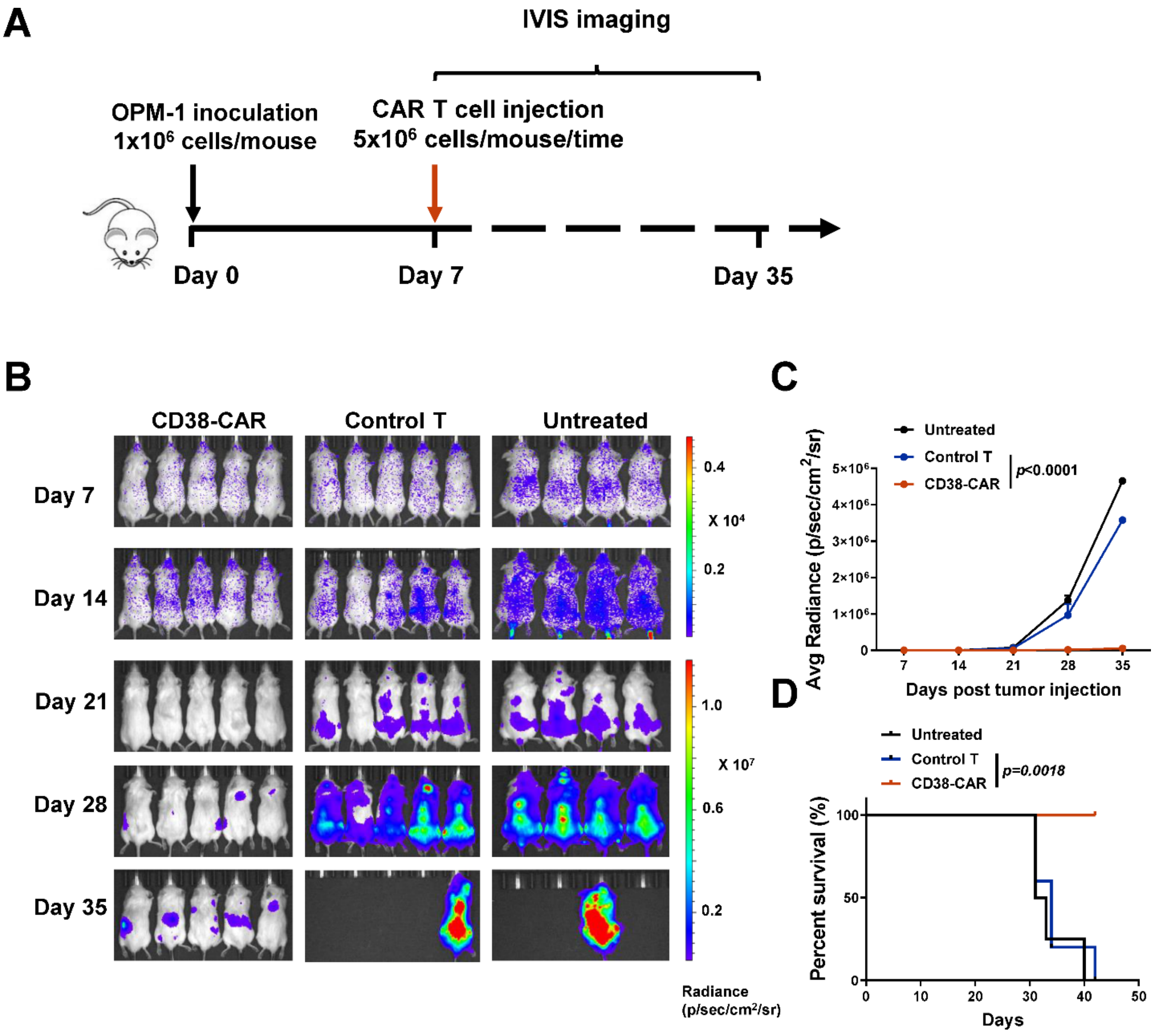

Supplemental Figure S2. Anti-myeloma function of CD38-CAR T cells *in vivo.*

(A) Schematic of CAR T cells treatment protocol. NSG mice were intravenously injected with 1 × 10^6 fLuc-transduced MM cells (OPM-1). After 7 days, the mice were treated with 5×10^6 of CD38-CAR T cells, control T cells, or remained untreated (n = 5 or 4). (B) A serial of representative bioluminescent imaging showing myeloma progression/regression in mice. (C) Kinetics of myeloma progression in mice treated with CD38-CAR T cells, control T cells, or remained untreated measured by bioluminescent imaging. (D) Survival curve for each experimental group.

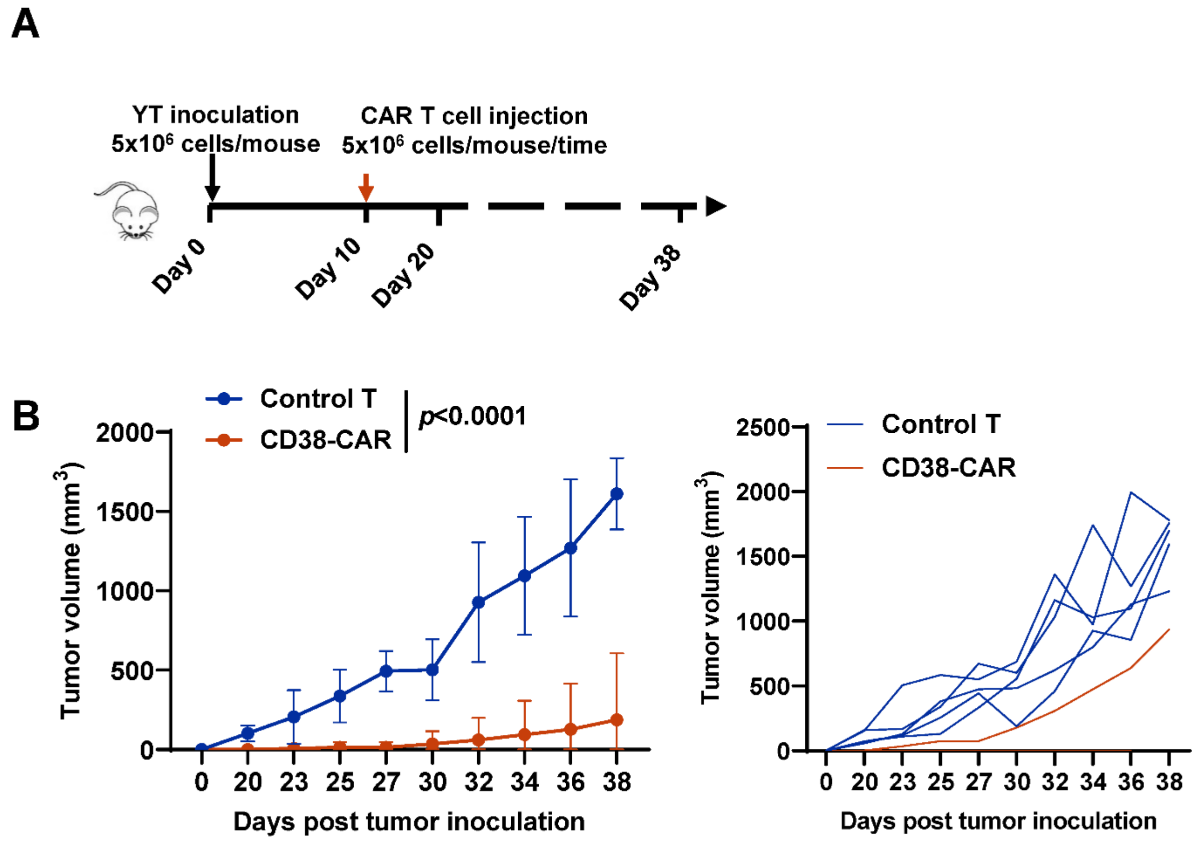

Supplemental Figure S3. CD38-CAR T cells inhibit NKTCL growth in the prophylactic xenograft model.

(A) Schematic of CAR T cells treatment protocol. NSG mice were subcutaneously injected with 5×10^6 NKTCL cells (YT). After 10 days, the mice were treated with 5×10^6 of CD38-CAR T cells or control T cells (n= 5). (B) Mean tumor growth kinetics for each treatment group.

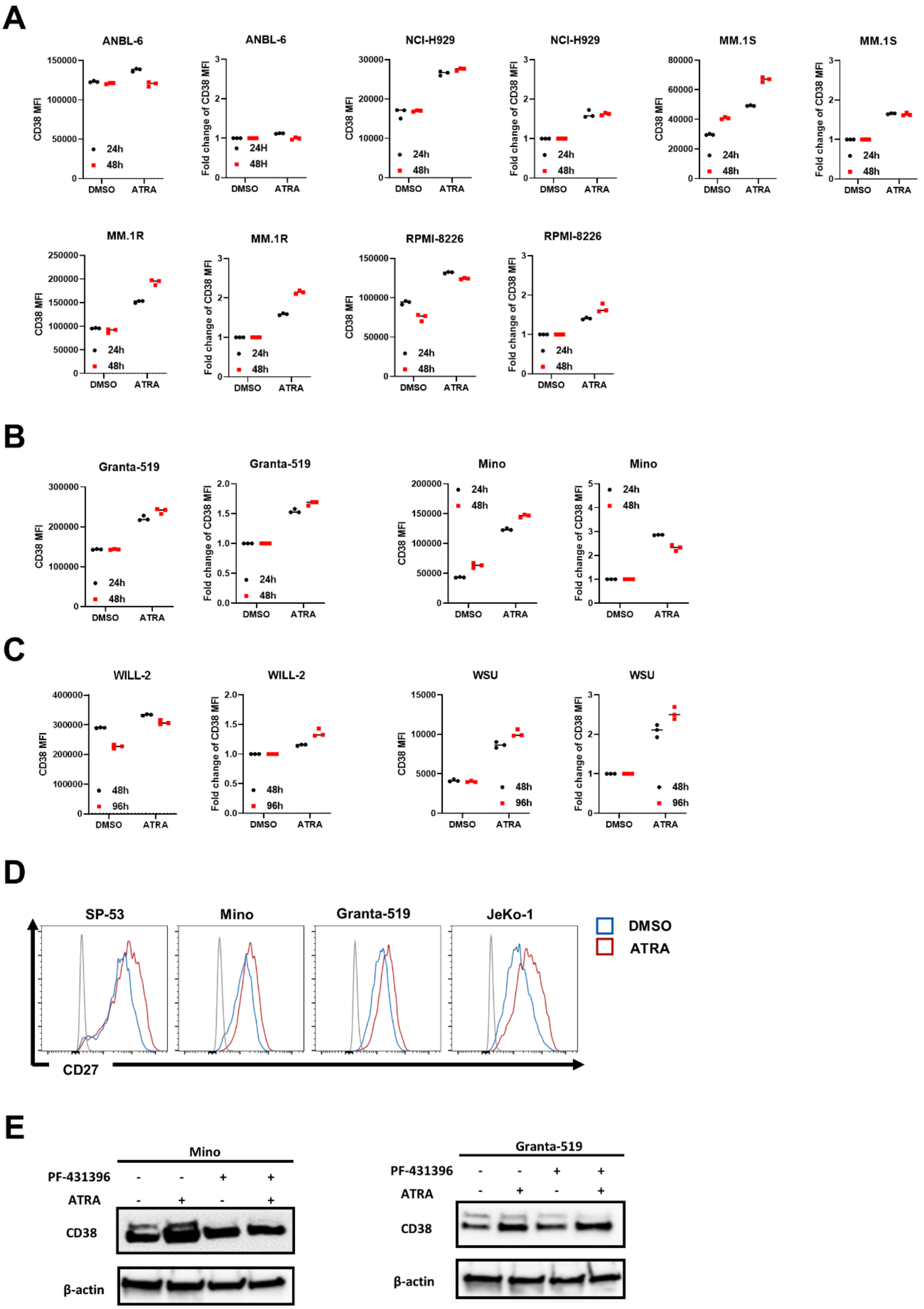
A
ANBL-6
NCI-H929
MM.1S
MM.1R
RPMI-8226
CD38 MFI
Fold change of CD38 MFI
24h
48h
DMSO
ATRA
B
Granta-519
Mino
C
WILL-2
WSU
96h
D
SP-53
JeKo-1
CD27
E
PF-431396
CD38
β-actin

Supplemental Figure S4. ATRA enhances the CD38 expression in MM, MCL, and DLBCL.

(A–C) CD38 expression in MM, MCL, or DLBCL with or without ATRA (10 nM) treatment by flow cytometry. (D) CD27 expression in MCL cell lines with or without ATRA (10 nM) treatment (JeKo-1, Granta-519, Mino and SP-53) by flow cytometry. (E) Mino and Granta-519 were treated with ATRA (10 nM) or PF-431396 for 48 hours. CD38 expression were evaluated by immunoblotting.

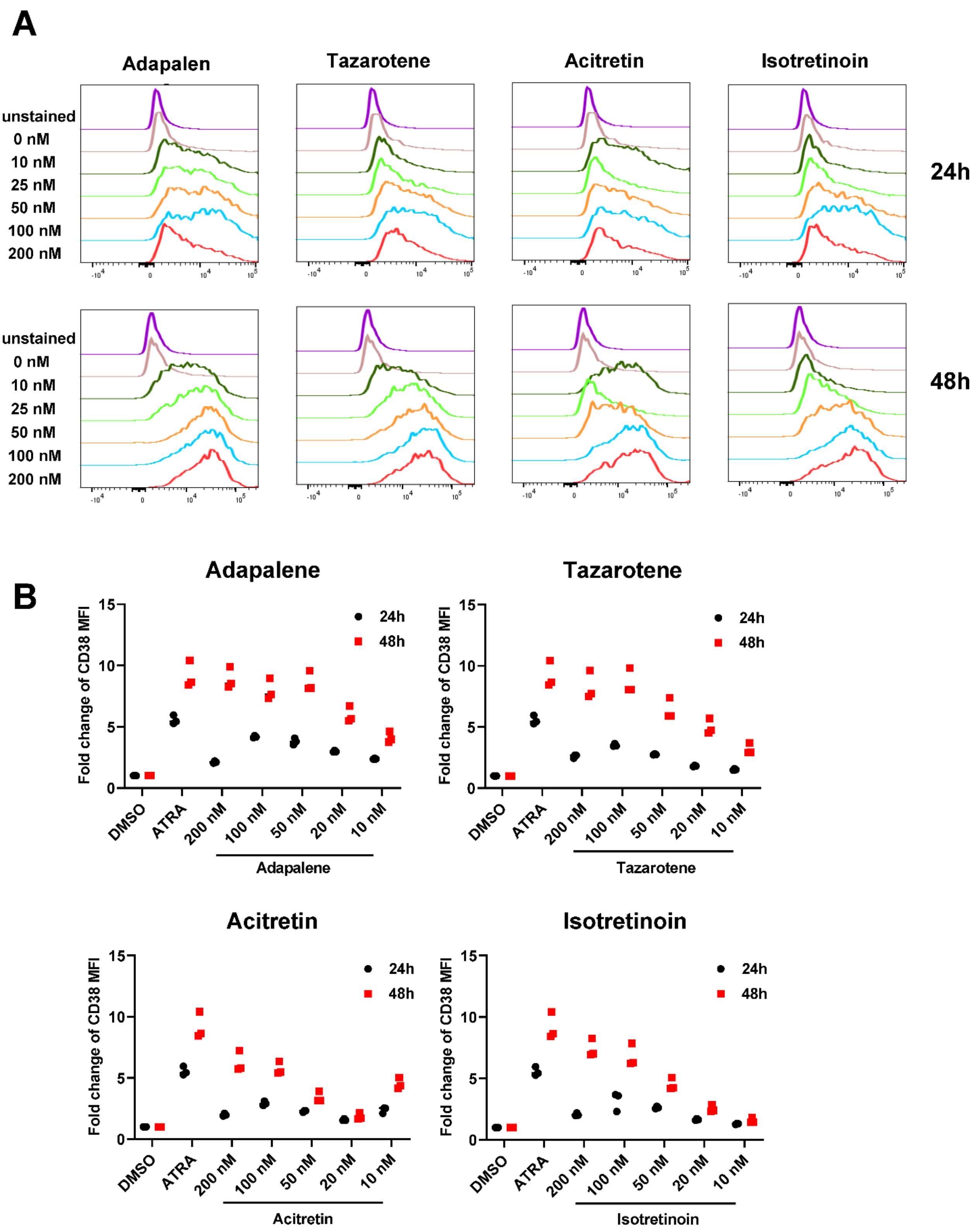

Supplemental Figure S5. Retinoids enhance the CD38 expression in variable degrees.

(A) SP-53 cells were treated with adapalene, tazarotene, acitretin, isotretinoin, or the vehicle control ranging from 10 to 200 nM for 24 or 48 h. (B) The fold change of median fluorescence intensity (MFI) of CD38 in the SP-53 cells after treatment of ATRA (10 nM) or four other retinoids in 24 or 48 h.

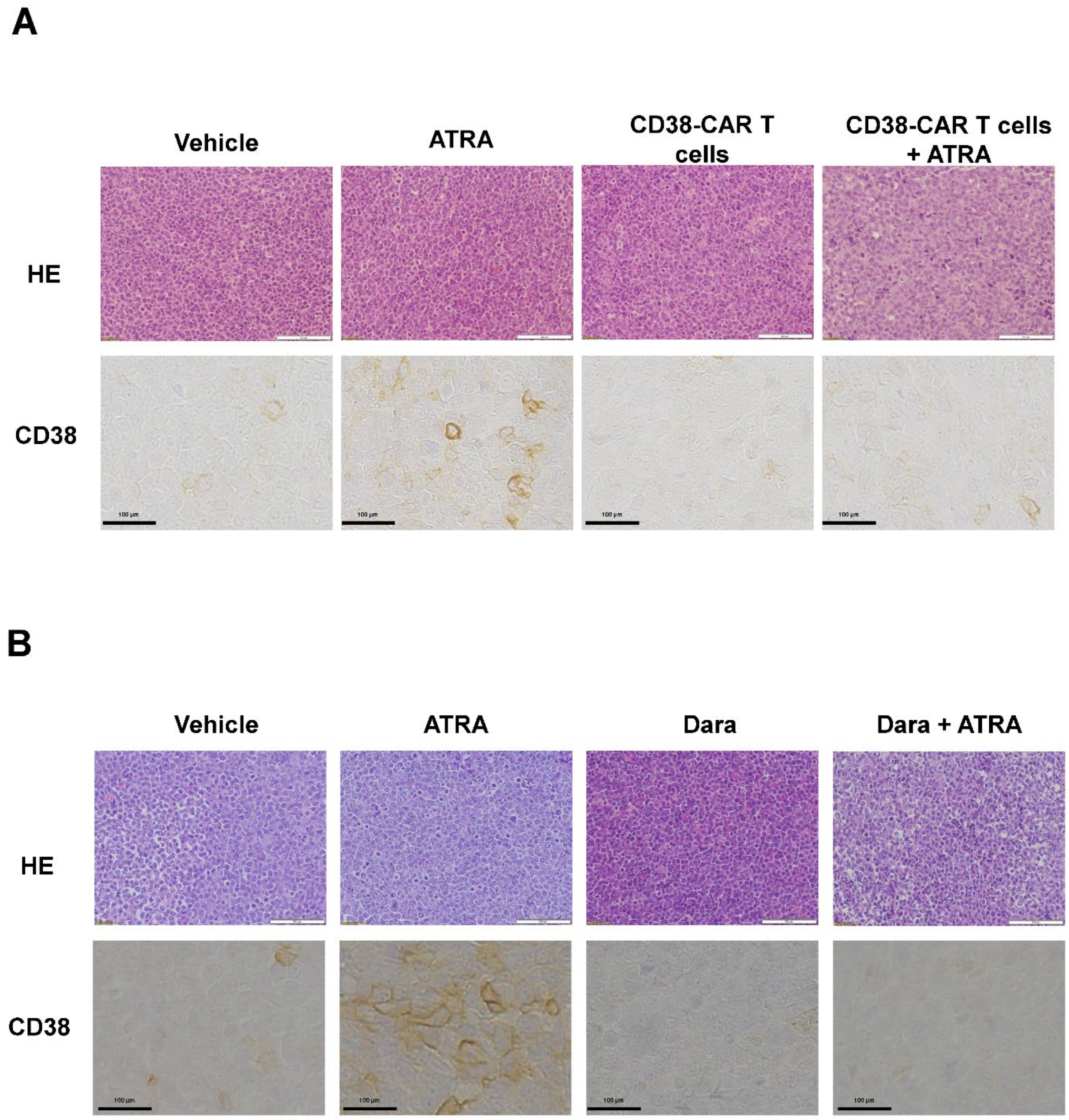

Supplemental Figure S6. Hematoxylin and eosin (H&E) and immunohistochemistry (IHC) staining for tumors from mice with ATRA or anti-CD38-based immunotherapy.

(A) Representative IHC images of indicated treatment SP-53 tumors for CD38. Scale bars, 100 μm; H&E staining of SP-53 tumors on day 22. Scale bars, 100 μm. (B) Representative IHC images of indicated treatment SP-53 tumors for CD38. Scale bars, 100 μm; H&E staining of SP-53 tumors on day 22. Scale bars, 100 μm.

Supplemental Table 1. Cell lines used in this study

Name	Cancer type	Medium	CD38 expression
RPCI-WM1	WM (NHL)	RPMI-1640	+
JeKo-1	MCL (NHL)	RPMI-1640	+
Granta-519		RPMI-1640	+
Mino		RPMI-1640	+
SP-53		RPMI-1640	+ (low)
MOLT-4	T-ALL	RPMI-1640	+
Jurkat		RPMI-1640	+ (low)
NK-92	NKTCL (NHL)	α-MEM +IL-2	+
HANK-1		RPMI-1640+IL-2	+
KHYG-1		RPMI-1640+IL-2	+
NK-YS		RPMI-1640+IL-2	+
YT		IMDM	+
SNK-6		RPMI-1640+IL-2	-
WILL-2	DLBCL (NHL)	RPMI-1640	+ (low)
WSU		RPMI-1640	+
MM.1S	MM	RPMI-1640	+
MM.1R		RPMI-1640	+
NCI-H929		RPMI-1640	+
OPM-1		RPMI-1640	+
RPMI-8226		RPMI-1640	+
KMS-12		RPMI-1640	+ (low)
ANBL-6		RPMI-1640	+
K562	CML	RPMI-1640	-
